# A Baicalin‐Based Functional Polymer in Dynamic Reversible Networks Alleviates Osteoarthritis by Cellular Interactions

**DOI:** 10.1002/advs.202410951

**Published:** 2025-01-22

**Authors:** Yili Yang, Qinxiao Hu, Qingfeng Shao, Yachen Peng, Bo Yu, Fangji Luo, Jiajing Chen, Chenhao Xu, Zhenyan Li, Manseng Tam, Zhenyu Ju, Ronghua Zhang, Feiyue Xing, Zhengang Zha, Huan‐Tian Zhang

**Affiliations:** ^1^ Department of Bone and Joint Surgery the First Affiliated Hospital of Jinan University Key Laboratory of Regenerative Medicine of Ministry of Education Jinan University Guangzhou Guangdong 510630 China; ^2^ Department of Immunobiology, College of Life Science and Technology Jinan University Guangzhou 510632 China; ^3^ Macau Medical Science and Technology Research Association Macao 999078 China; ^4^ Guangdong Provincial Key Laboratory of Traditional Chinese Medicine Informatization College of Pharmacy Jinan University Guangzhou Guangdong 510630 China; ^5^ Department of Immunobiology College of Life Science and Technology Jinan University Guangzhou Guangdong 510632 China; ^6^ Department of Immunobiology MOE Key Laboratory of Tumor Molecular Biology Jinan University Guangzhou 510632 China

**Keywords:** anti‐inflammation, Bai‐based polymer, glycolysis, osteoarthritis, Schiff‐base hydrogel

## Abstract

Osteoarthritis (OA) is increasingly recognized as a whole‐organ disease predominantly affecting the elderly, characterized by typical alterations in subchondral bone and cartilage, along with recurrent synovial inflammation. Despite the availability of various therapeutics and medications, a complete resolution of OA remains elusive. In this study, novel functional hydrogels are developed by integrating natural bioactive molecules for OA treatment. Specifically, baicalin (Bai) is combined with 2‐hydroxyethyl acrylate (HEA) to form a polymerizable monomer (HEA‐Bai) through esterification, which is subjected to reversible addition‐fragmentation chain transfer (RAFT) polymerization to produce Bai‐based polymer (P_m_). These macromolecules are incorporated into Schiff‐base hydrogels, which demonstrate excellent mechanical properties and self‐healing performance. Notably, the Bai‐based formulations are taken up by fibroblast‐like synoviocytes (FLSs), where they regulate glycolysis. Mechanistically, inhibition of yes‐associated protein 1 (YAP1) by the formulations suppressed the FLSs glycolysis and reduced the secretion of inflammatory factors, including interleukin 1β (IL‐1β), IL‐6, and IL‐8. Furthermore, the functional hydrogel (AG‐P_m_)‐OC, severing as a lubricant and nutrient, prolonged joint retention of Bai, thereby reducing cartilage degradation and synovial inflammation. Meanwhile, (AG‐P_m_)‐OC alleviated joint pain by targeting the YAP1 signaling and inhibiting macrophage recruitment and polarization. Taken together, this flavonoid‐based injectable hydrogel exhibits enhanced biocompatibility and efficacy against OA.

## Graphical abstract

This study reveals that novel baicalin (Bai) macromolecules based on esterification and reversible addition‐fragmentation chain transfer (RAFT) polymerization alleviate osteoarthritis (OA) progression by integrated into dynamic crosslinked networks. The formulations target the YAP1 signaling and inhibit macrophage polarization, effectively suppressing the glycolysis of fibroblast‐like synoviocytes (FLSs) and reducing the secretion of inflammatory factors, thereby decreasing cartilage degradation and synovial inflammation.

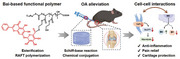

## 1 Introduction

Osteoarthritis (OA) is a chronic, degenerative disorder that primarily affects the elderly, with mechanisms that remain unclear.^[^ ^1^ ^]^ Recent studies have demonstrated that synovial cell‐cell interactions play a key role in initiating OA development and progression. For instance, fibroblast‐like synoviocyte cells (FLSs) can modulate macrophage metabolism, while inflammatory macrophages, in turn, regulate the proliferation and behavior of FLSs during OA‐related inflammation.^[^ ^2^ ^]^ Macrophage polarization toward the M1 phenotype is a hallmark of OA, and reprogramming M1 macrophage to the M2 phenotype presents a promising strategy to delay OA progression.^[^ ^3^ ^]^ Additionally, suppressing M1 macrophage recruitment may help reduce synovial inflammation.^[^ ^4^ ^]^ Previous research has revealed that yes‐associated protein 1 (YAP1) is significantly increased in OA and is crucial for FLSs‐macrophages interactions that drive synovial inflammation.^[^ ^5^ ^]^ Our recent work has shown that diabetic OA can be ameliorated by suppressing synovial glycolysis and macrophage infiltration via the YAP1 signaling,^[^ ^6^ ^]^ highlighting its potential in managing joint disease.

Baicalin (Bai), a flavonoid derived from the roots of *Scutellaria baicalensis*, exhibits a range of pharmacological activities, including anti‐inflammatory and antioxidant effects.^[^ ^7^ ^]^ However, its low hydrophilicity and lipophilicity pose challenges to its bioavailability, necessitating further studies to optimize its clinical applications.^[^ ^8^ ^]^ For OA therapy, Bai has been reported to ameliorate the apoptotic and catabolic phenotype of chondrocytes in articular cartilage.^[^ ^9^ ^]^ It also reduces oxidative stress levels by regulating mitochondrial succinate dehydrogenase, with reduced loss of glutamine synthetase.^[^ ^10^ ^]^ According to a previous study, Bai inhibits IL‐1β‐induced expression levels of inflammatory cytokines by blocking NF‐κB signaling, showing cartilage protection in OA mice.^[^ ^11^ ^]^ In addition, Bai can activate mitophagy in IL‐1β‐induced chondrocytes by inhibiting the PI3K pathway and activating the PINK1 pathway, thus reducing OA‐related chondrocytes damage.^[^ ^12^ ^]^ However, the mechanisms underlying Bai‐mediated OA protection remain not fully understood.

Injectable hydrogels have garnered significant interest in joint therapies due to their flexibility in filling irregular wound shapes.^[^ ^13^ ^]^ The biophysical properties of biomedical hydrogels can be fine‐tuned by adjusting precursor concentration, cross‐linking density, and gelation time, all of which affect cell behavior.^[^ ^14^ ^]^ Dynamic reversible hydrogels constructed by Schiff‐base reactions offer desirable mechanical properties and self‐healing performance, making them ideal candidates for use in joint disease.^[^ ^15^ ^]^ From the perspective of synovial inflammation in OA, hydrogels granted with anti‐inflammatory activity are particularly valuable. Versatile hydrogels have been utilized for anti‐inflammation by loading liposomes, flavonoids, and peptides.^[^ ^16^ ^]^ For instance, an electro‐assisted bio‐fabricated hydrogel containing naringin shows anti‐inflammatory effects and promotes osteogenic differentiation.^[^ ^17^ ^]^ However, small molecules have rarely been designed into macromolecules via chemical conjugation to achieve powerful therapeutic combinations.^[^ ^18^ ^]^ Therefore, the controlled release of bioactive molecules such as Bai, through strong chemical conjugation, holds promise for preventing the rapid leakage of targeted drugs.

**Scheme 1:**
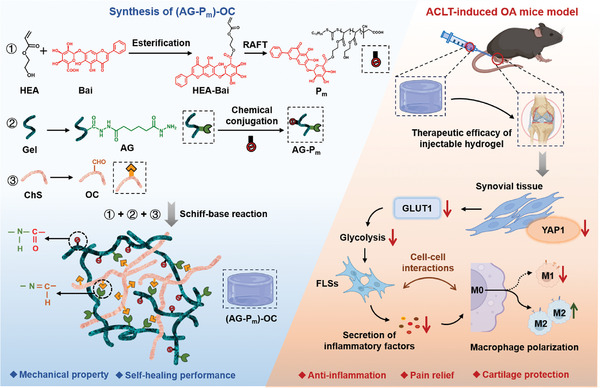
Schematic representation of the synthesis of HEA‐Bai, P_m_, AG, AG‐P_m_, OC, and (AG‐P_m_)‐OC and the investigation of the bioactivies of Bai‐based functional formulations.

In this work, we synthesized a Bai‐based bioactive polymer (P_m_) by combining reversible addition‐fragmentation chain transfer (RAFT) polymerization with esterification. P_m_ was subsequently conjugated to aminated gelatin (AG) and combined with oxidized chondroitin sulfate (OC) via a Schiff‐base reaction (**Scheme** **1**). The resulting reversible cross‐linked networks exhibited desired lubrication and nutrition functionalities, attributed to the natural precursors of the injectable hydrogels, chondroitin sulfate (ChS) and gelatin (Gel). The functional hydrogels ((AG‐P_m_)‐OC) demonstrated superior anti‐inflammatory activity and cartilage protection effects in both in vitro and in vivo models, introducing a novel paradigm for the treatment of degenerative joint disease, including OA.

## 2 Results

### 2.1 Synthesis and Characterization of Functionalized AG‐Pm

**Figure 1:**
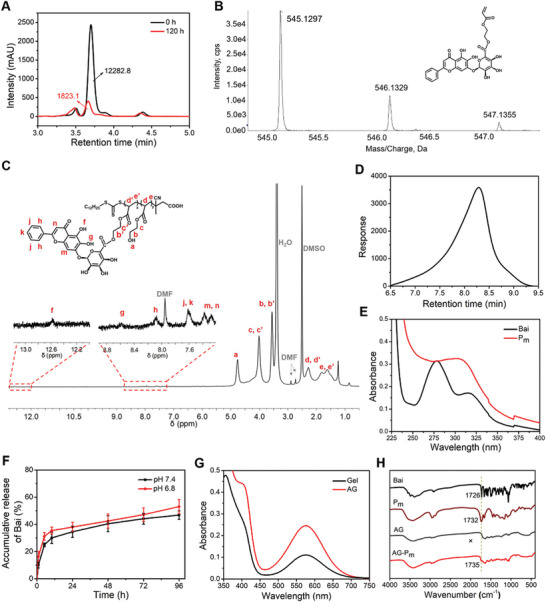
Characterization of Bai‐based monomer HEA‐Bai, macromolecular P_m_, and precursor AG‐P_m_. A) High‐performance liquid chromatography (HPLC) analysis showing the conversion of Bai to HEA‐Bai. B) High‐resolution mass spectrometry (HRMS) reveals the accurate molecular weight of HEA‐Bai. C) Hydrogen nuclear magnetic resonance (^1^H NMR) spectra of P_m_. D) Size exclusion chromatography (SEC) analysis demonstrating the structural uniformity of P_m_. E) UV–vis spectroscopy determining the Bai content in P_m_. F) In vitro cumulate release profile of Bai from P_m_ at pH 6.8 and 7.4. G) Ninhydrin assay results were analyzed by UV–vis spectroscopy. H) Fourier transform infrared spectroscopy (FT‐IR) spectra of Bai, P_m_, AG, and AG‐P_m_.

Bai possesses natural bioactivities due to its unique chemical structure, but its applications are hindered by rapid metabolism and poor bioavailability.^[^ ^19^ ^]^ To solve the problems, Bai was designated to undergo esterification by combining it with a hydrophilic monomer, HEA, to form a polymerizable monomer (HEA‐Bai), which was equipped with C = C double bonds to facilitate subsequent polymerization. Notably, the hydroxyl group in HEA and the carboxyl group in Bai enable to construction of HEA‐Bai while preserving the bioactivities of Bai.^[^ ^20^ ^]^ As shown in **Figure** **1**A, we have successfully obtained HEA‐Bai through esterification with a Bai conversion rate of up to 85%. Additionally, the accurate protonated molecule of HEA‐Bai was detected with an apparent signal at 545.1297 (C_26_H_24_O_13_, [M + H]^+^; calculated *m/z* = 545.1290) (Figure 1B). HEA‐Bai was then subjected to RAFT polymerization to obtain Bai‐based polymer (P_m_), to improve permeability through hydration backbones.^[^ ^21^ ^]^ The RAFT polymerization was mediated by 4‐cyano‐4‐[[(dodecylthio)carbonothioyl]thio]pentanoic acid (CDSPA), a thiocarbonylthio‐chain transfer agent, yielding narrowly distributed macromolecules.^[^ ^22^ ^]^ In Figure 1C, the macromolecular P_m_ was confirmed by peaks d, d′, e, and e′, corresponding to the backbone of HEA in P_m_, while peaks a, b, b′, c, and c′ were assigned to HEA pendants. Peaks g, h, j, k, m, and n indicated the presence of Bai in P_m_. As demonstrated in Figure 1D, the uniform curve of P_m_ showed the advantages of RAFT polymerization with reasonable dispersity (M_w_/M_n_) of 2.50 and a molecular weight (M_n_) of 36 200 g/mol. Moreover, the Bai content in P_m_ was determined to be 5.86% (Figure 1E) according to the standard curve of Bai (Figure S1, Supporting Information), which was close to the theoretical value of 8.7%, calculated by the mass ratio of Bai and P_m_. Compared to the neutral condition (pH 7.4), P_m_ exhibited a faster release of Bai at pH 6.8 (Figure 1F), consistent with previous reports that the ester bonds are more labile in acidic conditions, which might be beneficial for the OA joint environment.^[^ ^23^ ^]^

Gel, a product of collagen hydrolysis, is widely utilized in 3D cell culture and tissue engineering due to its abundance of amino acids.^[^ ^24^ ^]^ Next, the amination of Gel was conducted under mild conditions to promote the Schiff‐base reaction between the amine‐functionalized Gel (AG) and oxidated ChS (OC) bearing aldehyde groups.^[^ ^25^ ^]^ To verify the structure of the Gel, a signal at 1.56 ppm was assigned to alanine, while a signal at 0.83 ppm indicated the presence of valine and leucine (Figure S2, Supporting Information).^[^ ^26^ ^]^ Notably, the intensity of peaks a and b in AG was attributed to aminated modification by introducing adipic acid dihydrazide (ADH). Furthermore, the amount of free amino groups was determined by a ninhydrin assay, according to the standard curve of glycine (Figure S3, Supporting Information).^[^ ^26^ ^]^ Compared with Gel, the free amino groups in AG increased to 220%, representing a considerable increase in amino groups (Figure 1G). Subsequently, the carboxylic end‐capped P_m_ was grafted to AG to improve mechanical properties using ethyl‐dimethyl‐aminopropylcarbodiimide (EDC) and *N*‐hydroxy‐succinimide (NHS) as coupling agents. According to FT‐IR spectra, the C═O stretching vibration of AG‐P_m_ and P_m_ were observed at 1735 and 1732 cm^−1^, respectively, which indicated the presence of Bai in AG‐P_m_ (Figure 1H). Additionally, ChS, as a major component of glycosaminoglycan in cartilage tissue,^[^ ^27^ ^]^ was oxidized to OC to facilitate the Schiff‐base reaction by transforming hydroxyl groups into aldehyde groups.^[^ ^28^ ^]^ Of note, the degree of oxidation of ChS was calculated to be 35%, based on the integrals at peaks δ = 1.4 ppm and δ = 1.9 ppm, representing the methyl groups of *t*‐butyl moieties and the methyl group of ChS, respectively (Figure S4, Supporting Information). Collectively, well‐characterized AG‐P_m_ and OC are synthesized through a series of chemical conjugations.

### 2.2 Rheological Study and Degradation Behavior of Functional (AG‐Pm)‐OC

Injectable hydrogels have been widely utilized in regenerative medicine because of their minimally invasive injection capabilities to conform to irregular joint defects.^[^ ^29^ ^]^ Schiff‐base hydrogels have been reported to exhibit dynamic cross‐linking properties, enhanced mechanical strength, and self‐healing capabilities.^[^ ^15^, ^30^ ^]^ Herein, various ratio of precursors was applied to construct functional hydrogels, namely (AG‐P_m_)‐OC. Clearly, 3D networks were observed with loose configuration which might promote cell adhesion and communication (Figure S5, Supporting Information).^[^ ^31^ ^]^

**Figure 2:**
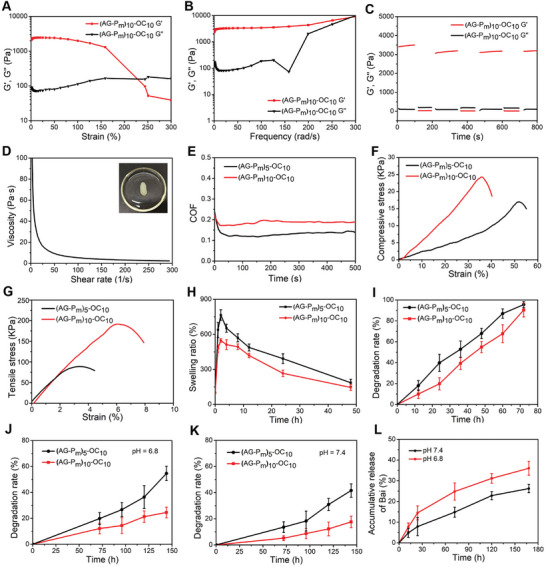
Rheological study, mechanical properties, and degradation of (AG‐P_m_)‐OC hydrogels. A) Strain amplitude sweep mode of (AG‐P_m_)_10_‐OC_10_. B) Frequency amplitude sweep mode of (AG‐P_m_)_10_‐OC_10_. C) Alternating strain amplitude sweep mode of (AG‐P_m_)_10_‐OC_10_. D) Shear‐thinning test of (AG‐P_m_)_10_‐OC_10_, inserted with a macroscopic image. E) Friction test of (AG‐P_m_)_10_‐OC_10_ and (AG‐P_m_)_5_‐OC_10_ under the loading of 5 N at 1 Hz. F) Compression test of (AG‐P_m_)_10_‐OC_10_ and (AG‐P_m_)_5_‐OC_10_. G) Tensile test of (AG‐P_m_)_10_‐OC_10_ and (AG‐P_m_)_5_‐OC_10_. H) Swelling behavior of (AG‐P_m_)_10_‐OC_10_ and (AG‐P_m_)_5_‐OC_10_. I) Enzymatic degradation of (AG‐P_m_)_10_‐OC_10_ and (AG‐P_m_)_5_‐OC_10_ with Collagenase Type I. J) Hydrolysis degradation of (AG‐P_m_)_10_‐OC_10_ and (AG‐P_m_)_5_‐OC_10_ at pH 6.8. K) Hydrolysis degradation of (AG‐P_m_)_10_‐OC_10_ and (AG‐P_m_)_5_‐OC_10_ at pH 7.4. L) In vitro cumulate release profile of Bai from (AG‐P_m_)_10_‐OC_10_ at pH 6.8 and pH 7.4.

To investigate the rheological performance of the functional hydrogels, we performed four sweep modes using a Malvern Kinexus Pro+ rheometer. As illustrated in **Figure** **2**A, the strain amplitude sweep mode of (AG‐P_m_)_10_‐OC_10_ showed a linear viscoelastic region ranging from 0% to 229% strain, with the storage modulus (G′) exceeding the loss modulus (G″). Similarly, (AG‐P_m_)_5_‐OC_10_ exhibited a linear viscoelastic region from 0% to 126% strain (Figure S6A, Supporting Information). Notably, the crossover point of (AG‐P_m_)_10_‐OC_10_ (227% strain) was higher than that of (AG‐P_m_)_5_‐OC_10_ (125% strain), which is attributed to the increased content of AG‐P_m_.^[^ ^32^ ^]^ These results indicate that a stronger cross‐linking density resulting from the amino groups on AG‐P_m_ can significantly enhance the mechanical properties of the hydrogels. Furthermore, the frequency‐dependent rheological behavior of the stable (AG‐P_m_)_10_‐OC_10_ revealed its solid‐like characteristics and abilities to maintain the original network structure ranging from 0.1 to 300 rad s^−1^, with G′ remaining higher than G″ (Figure 2B).^[^ ^28^ ^]^ While (AG‐P_m_)_5_‐OC_10_ displayed similar behavior, its moduli values were lower than those of (AG‐P_m_)_10_‐OC_10_, demonstrating that a higher AG‐P_m_ content improves the mechanical properties (Figure S6B, Supporting Information). Accordingly, (AG‐P_m_)_10_‐OC_10_ was selected for alternating strain sweep mode to evaluate the self‐healing properties, which are crucial for OA treatment.^[^ ^15b^ ^]^ The G′ value of (AG‐P_m_)_10_‐OC_10_ was higher than its G″ value at 1% strain with network integrity (Figure 2C). However, the G′ value rapidly decreased below the G″ value, signifying network disruption at 300% strain. Remarkably, (AG‐P_m_)_10_‐OC_10_ exhibited recovery behavior over three cycles, with the G′ value surpassing the G″ value as the strain decreased from 300% to 1%, demonstrating efficient self‐healing properties. Moreover, the injectability of (AG‐P_m_)_10_‐OC_10_ was evaluated by a shear thinning test, which revealed a decrease in viscosity across a range from 0.1 to 300 s^−1^ (Figure 2D). Rheological studies revealed that the mechanical strength of (AG‐P_m_)_10_‐OC_10_ was successfully improved by incorporating dynamic covalent bonds within the reversible network.

The destruction of joint structures in OA significantly alters the biomechanical properties and frictional properties of healthy articular cartilage which is featured with low friction and high compressive stiffness.^[^ ^33^ ^]^ The friction tests of (AG‐P_m_)_10_‐OC_10_ and (AG‐P_m_)_5_‐OC_10_ were investigated on a Rtec MFT‐5000 tribometer, where a glass ball mimicked the surface of articular cartilage under wet conditions and PBS solution (pH 6.8) served as a lubricant. The hydrogels were subjected to a 5 N load at 1 Hz. As shown in Figure 2E, (AG‐P_m_)_5_‐OC_10_ and (AG‐P_m_)_10_‐OC_10_ both displayed coefficient of friction (COF) as 0.13 and 0.18, respectively, lower than previously reported COF of articular cartilage (0.2).^[^ ^34^ ^]^ Furthermore, Figure 2F depicts the distinct of different crosslinked degrees on hydrogels’ comprehensive properties. (AG‐P_m_)_10_‐OC_10_ showed a higher compressive stress (24.3 KPa) than (AG‐P_m_)_5_‐OC_10_ (16.9 KPa). Tensile properties of the functional hydrogels were performed to assess mechanical properties comprehensively. Likewise, the tensile stress of (AG‐P_m_)_10_‐OC_10_ was higher than that of (AG‐P_m_)_5_‐OC_10_ (Figure 2G).

The unwanted swelling behavior of hydrogels can deteriorate cohesion and compromise the internal structure of the networks.^[^ ^35^ ^]^ (AG‐P_m_)_10_‐OC_10_ exhibited a lower swelling ratio than (AG‐P_m_)_5_‐OC_10_, attributed to a higher cross‐linking density of (AG‐P_m_)_10_‐OC_10_, reducing the uptake of free water molecules (Figure 2H).^[^ ^36^ ^]^ Besides, hydrogels may influence organic metabolism after degrading into cellular microenvironment regulating cell behaviors and phenotypes.^[^ ^37^ ^]^ As shown in Figure 2I, enzymatic degradation of the hydrogels revealed substantial degradation within 72 h, with the extent of degradation positively correlated with the duration. The degradation of (AG‐P_m_)_5_‐OC_10_ at pH 6.8 showed a higher degradability (55%) compared to (AG‐P_m_)_10_‐OC_10_ (25%) after 144 h, owing to its lower cross‐linking density (Figure 2J). In Figure 2K, the degradation of hydrogels mimicking physiological conditions at pH 7.4 appeared a similar behavior, albeit with a slightly slower degradation rate. This phenomenon stems from Schiff‐base binding and ester bonds in (AG‐P_m_)‐OC hydrogels being labile under acidic conditions, accelerating degradation.^[^ ^38^ ^]^ Additionally, sustained release of Bai from (AG‐P_m_)_10_‐OC_10_ was observed in Figure 2L. These findings offer a feasible approach to prolong the circulation of Bai and thus overcoming burst release by adjusting hydrogels degradability to sustain long‐term stability for OA treatment.

### 2.3 P_m_ Regulates FLSs’ Inflammatory via Suppressing Glycolysis

**Figure 3:**
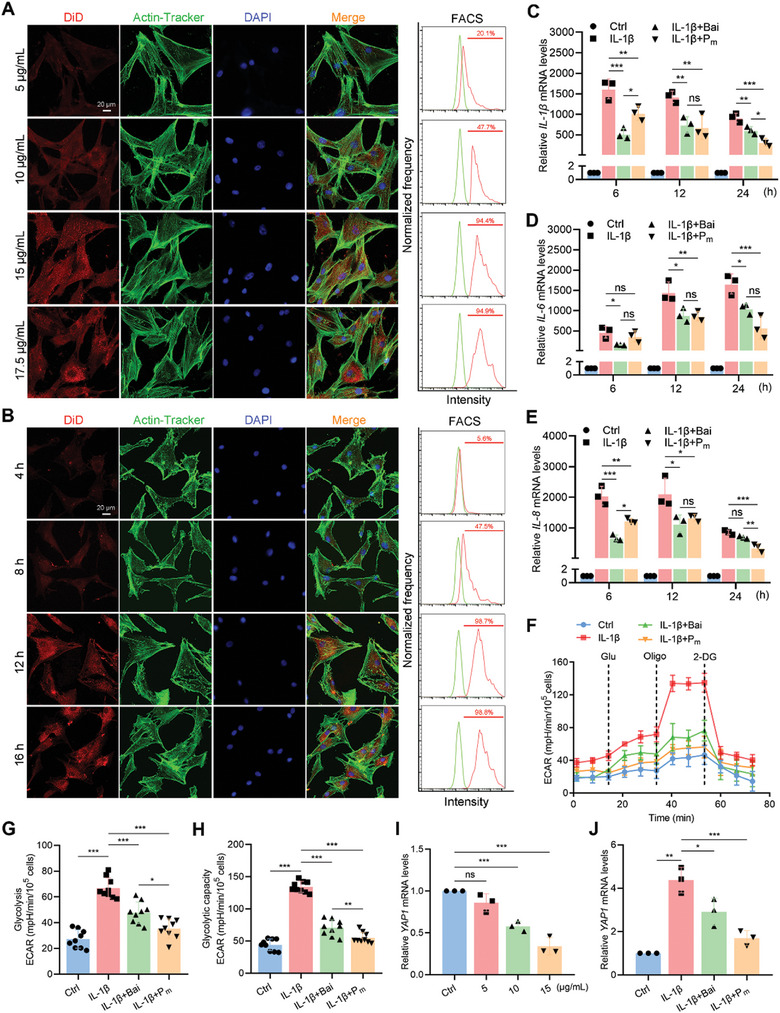
P_m_ regulates the secretion of inflammatory factors via YAP1‐mediated glycolysis in FLSs. A) Representative fluorescence images of FLSs treated with P_m_ at 5, 10, 15, and 17.5 µg mL^−1^ for 12 h. Scale bars = 20 µm. B) Representative fluorescence images of FLSs treated with P_m_ at 15 µg mL^−1^ for 4, 8, 12, and 16 h. Scale bars = 20 µm. C–E) Expression levels of *IL‐1β* (C), *IL‐6* (D), and *IL‐8* (E) in FLSs treated with Bai and P_m_ at 15 µg mL^−1^ for 6, 12, and 24 h. F–H) Seahorse metabolic flux measuring ECAR (F), glycolysis (G), and glycolytic capacity (H) in FLSs treated with Bai and P_m_ in the presence or absence of IL‐1β for 24 h. I) YAP1 expression levels in FLSs treated with P_m_ at 5, 10, and 15 µg mL^−1^ for 24 h. J) YAP1 expression levels in FLSs treated with Bai and P_m_ at 15 µg mL^−1^ for 24 h. Data are presented as mean ± SD. n.s.: not significant, ^*^ *p* < 0.05, ^**^ *p* < 0.01, and ^***^ *p* < 0.001. One‐way ANOVA for (C–E and G–J).

Next, bioactivities of the synthesized macromolecular P_m_ were evaluated using FLSs in vitro (Figure S7A, Supporting Information). First, cytotoxicity of P_m_ to FLSs was assessed using various concentrations (0, 5, 10, 15, 20, and 25 µg mL^−1^) corresponding to the equivalent amount of Bai in P_m_. P_m_ at 15 µg mL^−1^ showed no cytotoxicity against FLSs, compared to the control group, and remained well above the cytotoxicity threshold (70%) according to ISO 10993–5 (Figure S7B, Supporting Information).^[^ ^32^ ^]^ Next, cellular uptake of P_m_ (15 µg mL^−1^) by FLSs was examined by labeling the P_m_ with 1,1′‐dioctadecyl‐3,3,3′,3′‐tetramethylindodicarbocyanine (DiD). In details, FLSs were co‐cultured with different concentrations of P_m_ (5, 10, 15, and 17.5 µg mL^−1^) for 12 h and observed using confocal laser scanning microscopy (CLSM) (**Figure** **3**A). The fluorescent intensity gradually increased from 5 to 15 µg mL^−1^ and reached saturation at 15 µg mL^−1^, indicating that 15 µg mL^−1^ was an ideal concentration for cellular uptake at 12 h. Meanwhile, FLSs treated with P_m_ (15 µg mL^−1^) were observed at 4, 8, 12, and 16 h. As shown in Figure 3B, 12 h was the optimal interval for FLSs to absorb P_m_. The results were also validated by fluorescence‐activated cell sorting (FACS) (Figure 3A,B).

Interleukin 1β (IL‐1β), IL‐6, and IL‐8 belonging to proinflammatory cytokines have been reported to accelerate OA development via activation of NF‐*κ*B signaling pathway.^[^ ^39^ ^]^ Thus, the anti‐inflammatory activity of P_m_ (15 µg mL^−1^) was evaluated in IL‐1β‐activated FLSs by detecting the mRNA expression levels of *IL‐1β*, *IL‐6*, and *IL‐8* at different intervals (Figure 3C–E). Notably, the Bai group exhibited a better anti‐inflammatory effect than the P_m_ group during the first 6 h, while the suppression of levels of *IL‐1β*, *IL‐6*, and *IL‐8* in the P_m_ group surpassed the Bai group at 24 h (Figure 3C–E). The observation could be explained by the rapid absorption of Bai at the earlier stage, whereas, the controlled release of Bai from P_m_ prolonged its circulation time, thereby enhancing its anti‐inflammatory activity.

Recently, we have demonstrated that enhanced glycolysis in FLSs plays a fundamental role in energy production and subsequent secretion of inflammatory factors.^[^ ^6^ ^]^ Next, the effect of P_m_ on glycolysis in FLSs was investigated. FLSs treated with IL‐1β showed the highest extracellular acidification rate (ECAR) (Figure 3F), indicating inflammation did enhance cell glycolysis and glycolytic capacity. However, P_m_ significantly suppressed glycolysis in FLSs compared to Bai and the control group (Figure 3F–H). Mitochondrial activity, as determined by oxygen consumption rate (OCR), is also crucial for the health and function of the synovium in OA. OCR experiments denoted that IL‐1β‐stimulated FLSs showed a maximal decrease in the OCR level, and then it slightly rebounded after different treatments with Bai or P_m_ (Figure S8, Supporting Information). Since YAP1 is demonstrated to regulate the glycolysis of FLSs Bai is also required for coordinating glycolysis via suppression of hypoxia‐inducible factor 1 subunit alpha (HIF‐1α) and increased number of mitochondria and DNA content.^[^ ^6^, ^40^ ^]^ Therefore, we pursued the possible connections between Bai and YAP1 in the content of glycolysis. Epidermal growth factor receptor (EGFR) can activate PI3K and PDK1, thus inhibiting the Hippo pathway for YAP1 activation.^[^ ^6^, ^41^ ^]^ Our results using molecular docking and Western Blotting (WB) did find that YAP1 was a potential downstream target of Bai (Figures S9 and S10, Supporting Information). In support of this, P_m_ dose‐dependently decreased the *YAP1* mRNA levels, compared to that of the control and Bai alone (Figure 3I,J). These results together suggest that P_m_ could be promising macromolecules for regulating the expression levels of inflammatory cytokines through glycolysis modulation via YAP1.

### 2.4 Prolonged Joint Retention and Biodistribution of Bai‐Based Formulations

**Figure 4:**
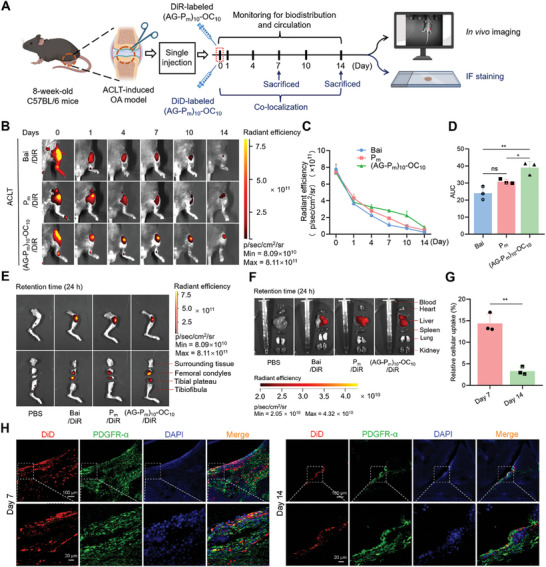
Joint retention, biodistribution, and co‐localization of Bai‐based formulations. A) Illustration of OA mice model which undergoes a series of treatments at different times and subsequent analysis. B) Representative images showing the time course of radiant efficiency in OA knee joints following a 10‐day intra‐articular injection of DiD‐labeled Bai, P_m_, and (AG‐P_m_)_10_‐OC_10_. C) Quantitative analysis of radiant efficiency in (B). D) Quantitative analysis of AUC in (C). E) Biodistribution of Bai‐based formulations in OA knee joints 24 h after a single injection. F) Biodistribution of Bai‐based formulations in major organs of OA mice 24 h after a single injection. G,H) Quantitative analysis of relative cellular uptake (G) and representative image of the fluorescence intensity (H) of DiD‐labeled (AG‐P_m_)_10_‐OC_10_ in OA knee joints, 7 and 14 days after a single injection. Scale bars = 100 and 20 µm. Data are presented as mean ± SD. n.s.: not significant, ^*^ *p* < 0.05, and ^**^ *p* < 0.01. Student's *t*‐test for (G) and one‐way ANOVA for (D).

The sustainable release of the drug in the joint cavity is crucial for the therapeutic efficiency of OA.^[^ ^32^ ^]^ Therefore, we investigated the retention and biodistribution of Bai‐based formulations using anterior cruciate ligament transection (ACLT)‐induced OA mice model. Bai, P_m_, and (AG‐P_m_)_10_‐OC_10_ were chemically tagged with 1,1‐dioctadecyl‐3,3,3,3‐tetramethylindotricarbocyanine iodide (DiR) and monitored by an in vivo imaging system (IVIS) (**Figure** **4**A). The fluorescence intensity in each group was initially similar and gradually decreased over time (Figure 4B). After 10 days, the remaining fluorescence intensity of the (AG‐P_m_)_10_‐OC_10_ group (2.24 × 10^11^ p sec^−1^ cm^−2^ sr^−1^) was higher than that of the Bai and P_m_ groups (Figure 4B). The area under the curve (AUC) of radiant efficiency indicated that the (AG‐P_m_)_10_‐OC_10_ group also had the best joint retention in OA mice (Figure 4C). By 14 days, the fluorescent signal was barely detectable in the Bai group but remained evident in the P_m_ and (AG‐P_m_)_10_‐OC_10_ group. These results suggest that macromolecules and crosslinked networks could prolong the retention of injectable formulations, overcoming the rapid clearance of small molecules in the joint of OA mice.^[^ ^42^ ^]^ The above findings reveal that (AG‐P_m_)_10_‐OC_10_ is a promising candidate for biomaterials, as its sustained retention enhances bioavailability and reduces the frequency of administration in OA treatment.^[^ ^43^ ^]^

In vivo, the biodistribution and toxicity of Bai‐based formulations should be clarified. To this end, OA mice were observed 24 h after a single injection of (AG‐P_m_)_10_‐OC_10_/DiR, P_m_/DiR, or Bai/DiR. Fluorescence imaging revealed that the hydrogel was localized in the femoral condyle and tibial plateau, yet it was then metabolized in the liver (Figure 4E,F), likely due to the clearance mechanisms involving dispersion in synovial fluid, capillary absorption, lymph drainage, excretion, and liver detoxification.^[^ ^44^ ^]^ To further determine the uptake of (AG‐P_m_)_10_‐OC_10_ in vivo, we performed immunofluorescence (IF) staining on the knee joint sections with a typical fibroblast marker, PDGFR‐α. PDGFR‐α stained cells (green) were found to co‐localize with the DiD‐labeled (AG‐P_m_)_10_‐OC_10_ cells (red), demonstrating an efficient targeting selectivity of (AG‐P_m_)_10_‐OC_10_ to FLSs (Figure 4G,H). These results together suggest that Bai‐based formulations could prolong joint retention and exhibit superior targeting selectivity to synovial tissue.

### 2.5 (AG‐P_m_)_10_‐OC_10_ Mitigates OA Progression and Synovial Inflammation

**Figure 5:**
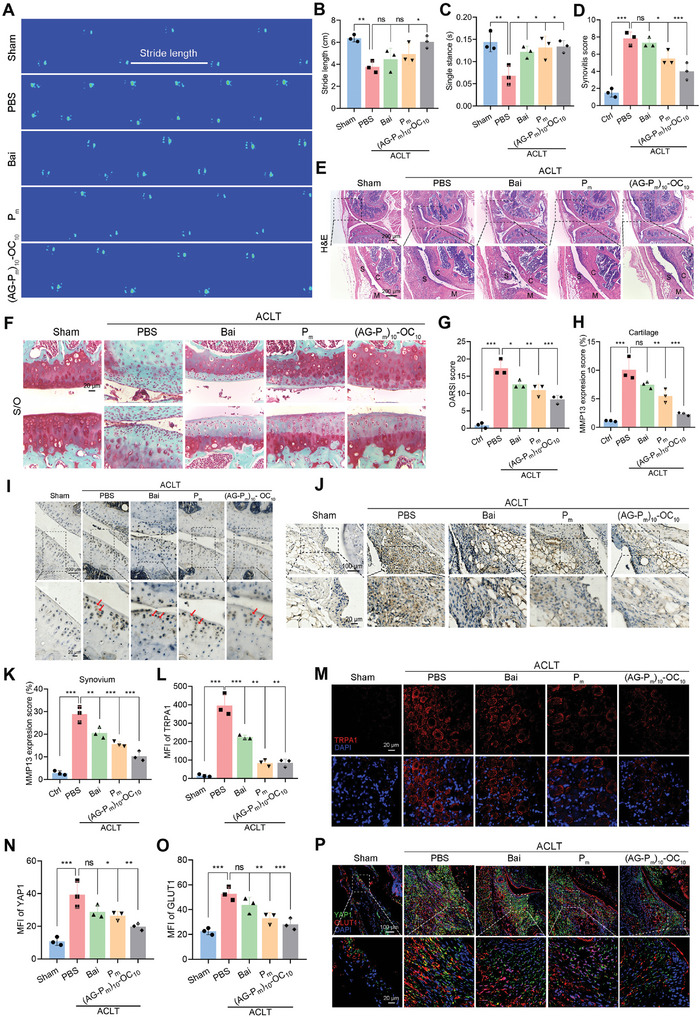
Bai‐based formulations mitigate OA progression via the YAP1/GLUT1 axis. A–C) Gait analysis of OA mice for footprints (A), stride length (B), and single stance (C), treated with Bai‐based formulations after six‐week ACLT surgery. D,E) Synovitis score (D) and representative H&E staining images (E) of OA knee joints following treatments with Bai, P_m_, and (AG‐P_m_)_10_‐OC_10_ for 6 weeks. Scale bars = 200 µm. F,G) Representative S/O staining images (F) and OARSI score (G) of knee joint severity following treatments with Bai, P_m_, and (AG‐P_m_)_10_‐OC_10_ for 6 weeks. Scale bars = 20 µm. H,I) Quantitative analysis (H) and representative immunohistochemical (IHC) images (I) of MMP13 in cartilage. Scale bars = 100 and 20 µm. J,K) Representative IHC images (J) and quantitative analysis (K) of MMP13 in synovial tissues. Scale bars = 100 and 20 µm. L,M) Quantitative analysis of TRPA1 fluorescence intensity (L) and representative fluorescence images of TRPA1 (red) in DRG tissues (J) following treatments with Bai, P_m_, and (AG‐P_m_)_10_‐OC_10_ for 6 weeks. Scale bars = 20 µm. N–P) Quantitative analysis of YAP1 (N) and GLUT1 (O) and representative fluorescence images (P) of YAP1 (green) and GLUT1 (red) following treatments with Bai, P_m_, and (AG‐P_m_)_10_‐OC_10_ for 6 weeks. Scale bars = 100 and 20 µm. Data are presented as mean ± SD. n.s.: not significant, ^*^ *p* < 0.05, ^**^ *p* < 0.01, and ^***^ *p* < 0.001. One‐way ANOVA for (B,C,D,G,H,K,L,N,O).

Gait analysis is a useful tool to investigate behavioral changes in preclinical OA models. Therefore, we applied the gait analysis to evaluate the role of Bai‐based formulations in alleviating joint pain. As shown in **Figure** **5**A–C, the footprints, hindlimb stride length, and single stance were apparently improved in the (AG‐P_m_)_10_‐OC_10_ group, compared with those in the PBS group. Next, histological analysis of knee joints was conducted on OA mice to assess biosafety and the therapeutic effects of the newly developed formulations. Treatments with the formulations in OA mice revealed no signs of necrosis or inflammatory infiltrates in the major organs (heart, liver, spleen, lung, and kidney), indicating the good biocompatibility of (AG‐P_m_)_10_‐OC_10_ (Figure S11, Supporting Information). Then, the synovitis and cartilage degeneration were analyzed by hematoxylin‐eosin (H&E) and safranin O/fast green (S/O) staining. Of note, the PBS group exhibited apparent synovial thickening, with an average synovitis score of 7.7 (Figure 5D,E). In contrast, (AG‐P_m_)_10_‐OC_10_ effectively reduced the synovial inflammation, reducing the score to 4.0, which outperformed Bai (score of 7.0) and P_m_ (score of 5.3). Consistently, cartilage lesions were observed in the PBS group with an Osteoarthritis Research Society International (OARSI) score of 7.8 (Figure 5F,G). However, the (AG‐P_m_)_10_‐OC_10_ group demonstrated a significant attenuation of cartilage degeneration, lowering the score to 4.0. Since MMP13 is a hallmark of OA, its expression was closely monitored to evaluate the progression and treatment effectiveness of OA.^[^ ^45^ ^]^ Low expression levels of MMP13 induce a plenitude of aggrecan and collagen II, reflecting a healthy metabolic state of articular cartilage.^[^ ^46^ ^]^ In agreement with a previous study, ACLT surgery led to a high expression level of MMP13 in both the cartilage (Figure 5H,I) and synovium (Figure 5J,K) in the PBS group, with the sham group serving as a negative control. However, treatment with (AG‐P_m_)_10_‐OC_10_ remarkably downregulated the expression of MMP13, outperforming the P_m_ and Bai groups.

TRPA1 has been recognized as a crucial chemical and mechanical pain sensor in response to inflammation and ACLT surgery.^[^ ^47^ ^]^ We have recently found that OA mice exhibited a higher expression level of TRPA1 in the dorsal root ganglia (DRG).^[^ ^44^ ^]^ Therefore, we explored whether (AG‐P_m_)_10_‐OC_10_ can regulate the TRPA1 expression in the DRG. In comparison to the PBS group, (AG‐P_m_)_10_‐OC_10_ treatment significantly decreased the TRPA1 expression in the DRG, indicating its superior effect on pain relief (Figure 5L,M). GLUT1‐driven synovial glycolysis has been linked to persistent joint inflammation and subsequent pain;^[^ ^48^ ^]^ then, the possible role of (AG‐P_m_)_10_‐OC_10_ in alleviating OA symptoms was explored. Results revealed a significant decrease of the expression of GLUT1 to PDGFR‐α which is increased in thickened OA synovium, in the treatments with Bai, P_m_, and (AG‐P_m_)_10_‐OC_10_ when compared to the PBS group (Figure S12, Supporting Information). The YAP1/GLUT1 signaling is demonstrated to increase significantly in OA and metabolic arthritis, leading to synovial inflammation.^[^ ^6^ ^]^ Herein, we found that (AG‐P_m_)_10_‐OC_10_ caused a simultaneous suppression of YAP1 and GLUT1 expression (Figure 5N–P). Collectively, (AG‐P_m_)_10_‐OC_10_ is a potent strategy for combating OA by inhibition of synovial inflammatory response.

### 2.6 (AG‐P_m_)_10_‐OC_10_ Protects Against OA via Modulation of Macrophages Polarization Both In Vitro and In Vivo

**Figure 6:**
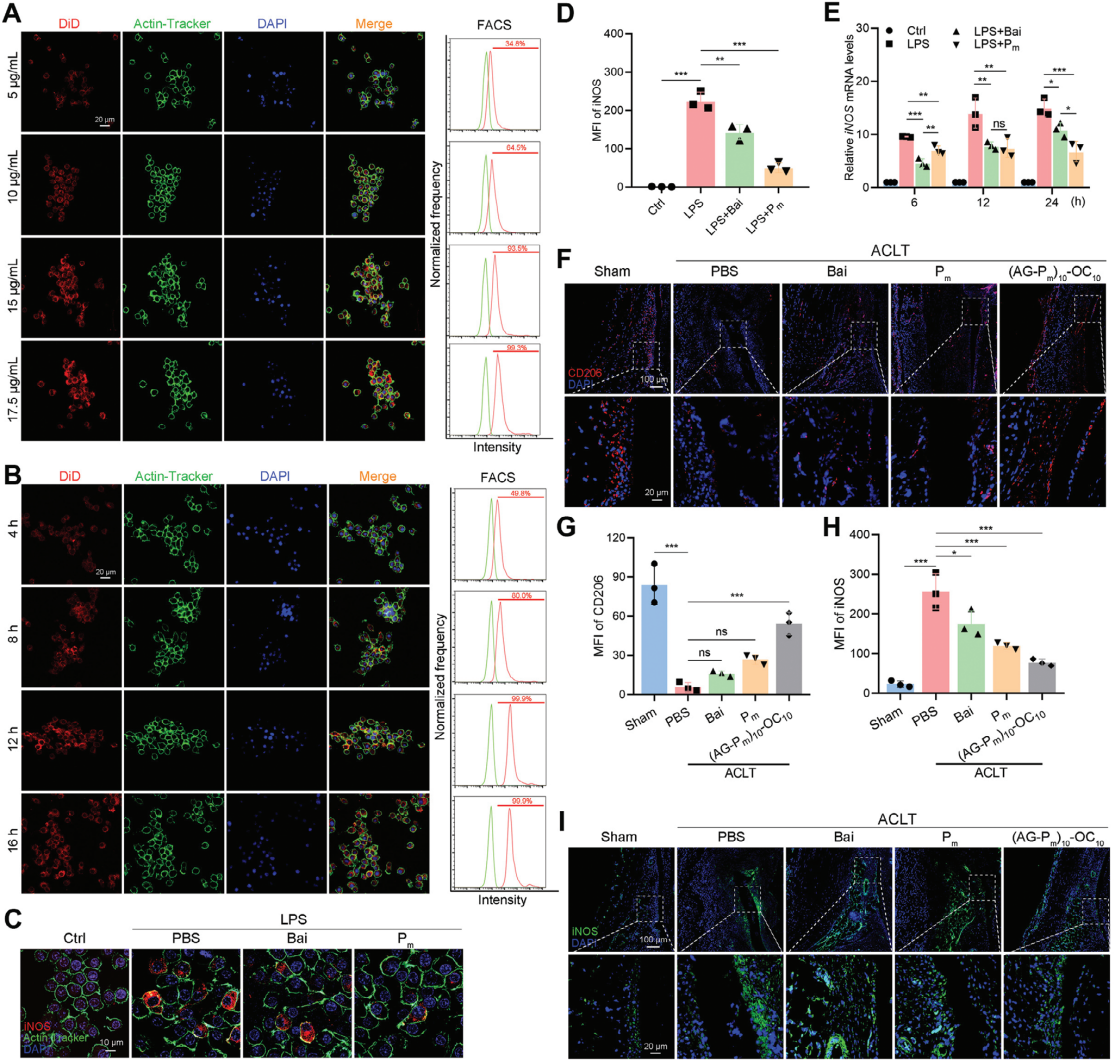
Bai‐based formulations regulate macrophage polarization. A) Representative fluorescence images of RAW264.7 cells treated with P_m_ at 5, 10, 15, and 17.5 µg mL^−1^ for 12 h. Scale bars = 20 µm. B) Representative fluorescence images of RAW264.7 cells treated with P_m_ at 15 µg mL^−1^ for 4, 8, 12, and 16 h. Scale bars = 20 µm. C,D) Representative fluorescence images of iNOS in RAW264.7 cells (C) and quantitative analysis of MFI of iNOS (D) after Bai or P_m_ treatments for 24 h (15 µg mL^−1^). Scale bars = 10 µm. E) Expression levels of *iNOS* in RAW264.7 cells treated with Bai and P_m_ at 15 µg mL^−1^ for 6, 12, and 24 h. F,G) Representative fluorescence images of CD206 (red) expression in synovial tissues (F) and quantitative analysis of MFI of CD206 (G) following treatments with Bai, P_m_, and (AG‐P_m_)_10_‐OC_10_ for 6 weeks. Scale bars = 20 µm. H,I) Quantitative analysis of MFI of iNOS (H) and representative fluorescence images of iNOS (green) expression in synovial tissues (I) following treatments with Bai, P_m_, and (AG‐P_m_)_10_‐OC_10_ for 6 weeks. Scale bars = 100 and 20 µm. Data are presented as mean ± SD. n.s.: not significant, ^*^ *p* < 0.05, ^**^ *p* < 0.01, and ^***^ *p* < 0.001. One‐way ANOVA for (D,E,G,H).

Increasing evidence has highlighted the critical role of macrophage recruitment and polarization in initiating OA phenotype.^[^ ^49^ ^]^ To explore this further, the mechanism of P_m_ was investigated using RAW264.7 cells. First, P_m_ showed no cytotoxicity to the RAW264.7 cells across a concentration range of 0–15 µg mL^−1^ (Figure S7C, Supporting Information). Similar to the observation in FLSs, DiD‐labeled P_m_ was taken up by RAW264.7 cells via a dose‐ and time‐dependent manner (**Figure** **6**A,B). Meanwhile, LPS‐induced RAW264.7 cells were stained with iNOS (a marker of M1 macrophages). Compared with the other groups, the P_m_ group showed a significant decrease in the expression level of iNOS (Figure 6C,D). Next, P_m_ also downregulated the *iNOS* mRNA expression level by quantitative Real‐time PCR (qRT‐PCR) (Figure 6E). In support of this, in vivo, results revealed a high expression level of CD206 (a marker of M2 macrophages) in the (AG‐P_m_)_10_‐OC_10_ group, compared to the other experimental groups (Figure 6F,G). Conversely, the expression of iNOS was decreased in the (AG‐P_m_)_10_‐OC_10_ group, indicating that (AG‐P_m_)_10_‐OC_10_ might serve as an effective therapeutic strategy for OA by regulating the macrophage polarization (Figure 6H,I). Overall, the Bai‐based functional formulations have demonstrated sustained OA therapeutic effects and it is a promising potential for long‐term development in biomedical applications.

## 3 Discussion

Osteoarthritis (OA), a chronic inflammatory disease, involves both synovial inflammation and cartilage degeneration.^[^ ^50^ ^]^ The complexity and heterogeneity of OA have led to an insufficiency of effective treatments in clinical practice.^[^ ^51^ ^]^ Therapies targeting synovial inflammation have shown significant success, as cell‐cell interactions within the synovium play a key role in triggering synovitis.^[^ ^6^ ^]^ Synovial mast cells are studied to modulate the pathogenesis of inflammatory arthritis by regulating protease‐activated receptor 2 (PAR‐2), assessed prior to OA treatment.^[^ ^52^ ^]^ Increasingly evidence holds the viewpoint that focusing on low‐grade and chronic inflammation can potentially modify OA progression.^[^ ^53^ ^]^ Our previous work demonstrated that OA can be moderately eased by targeting YAP1‐mediated glycolysis in FLS and subsequently, the macrophage polarization.^[^ ^6^, ^44^ ^]^

As a natural bioactive flavonoid, baicalin (Bai) is able to suppress the secretion of inflammatory factors and scavenge reactive oxygen species (ROS), associated with antioxidant and anticancer properties.^[^ ^54^ ^]^ Bai has been shown to alleviate OA‐related inflammatory injury by inhibiting the NF‐*κ*B signaling pathway through downregulation of miR‐126, a diagnostic marker of OA.^[^ ^55^ ^]^ Despite significant progress in the use of herbal small molecules for OA treatment, challenges such as rapid clearance and poor water solubility remain.^[^ ^56^ ^]^ In this study, we synthesized functional macromolecules based on Bai. Specifically, Bai underwent esterification with an acrylic monomer to form polymerizable HEA‐Bai. HEA‐Bai was then subjected to reversible addition‐fragmentation chain transfer (RAFT) polymerizations. RAFT polymerization has revolutionized polymer science by enabling the synthesis of well‐defined polymers with high‐end‐group fidelity, tunable molecular weight, and precise architecture.^[^ ^57^ ^]^ The resulting Bai‐based polymer (P_m_) exhibited a narrow molecular weight distribution, attributed to the advantages of RAFT polymerizations.

Injectable hydrogels with self‐healing performance offer distinct advantages over traditional biomaterials, particularly promoting tissue regeneration due to their viscoelastic and diffusive nature.^[^ ^58^ ^]^ Inspired by the benefits of injectable hydrogels, this study fabricates dynamic reversible hydrogels with enhanced mechanical properties through Schiff‐base reaction. The key to our approach is to exploit the carboxylic end‐capped P_m_ grafting onto aminated gelatin (AG) via amide linkages to form AG‐P_m_. Notably, the chemical conjugation between P_m_ and AG provides a strong connection, which is highly effective in preventing premature drug leakage.^[^ ^59^ ^]^ Additionally, strategies for cartilage protection, such as providing lubrication and nutrition, must also be emphasized, as they are crucial components of effective OA treatment. Herein, oxidized chondroitin sulfate (OC), derived from chondroitin sulfate—a key nutrient‐providing and lubricating component of synovial fluid, was prepared to introduce more aldehyde groups, facilitating the Schiff‐base reaction. The resulting covalently bound hydrogel, (AG‐P_m_)_10_‐OC_10_, exhibits ideal degradability, attributed to the ester bonds in P_m_ and the natural polymer precursors, which are triggered under acidic and enzymatic conditions.

Joint inflammation in OA is often initiated by cytokine networks between FLSs and macrophages, emphasizing the critical role of cell‐cell interactions in reprogramming OA pathology.^[^ ^60^ ^]^ This current work developed Bai‐based formulations and explored their potential mechanism for OA treatment. YAP1 is crucial for regulating tissue development and homeostasis, and it also supports chondrocyte proliferation through endochondral ossification and bone repair.^[^ ^61^ ^]^ P_m_ has been demonstrated to suppress the expression levels of inflammatory factors and glycolysis of FLSs, as confirmed by metabolomics data, with a reduced extracellular acidification rate (ECAR) and glycolytic capacity via the YAP1/GLUT1 signaling axis. Furthermore, severe pain and dysfunction, common in OA progression, are part of an irreversible pathological process.^[^ ^62^ ^]^ The (AG‐P_m_)_10_‐OC_10_ hydrogel was effective in relieving pain in the OA model by decreasing TRPA1 expression and delaying OA progression, as evidenced by decreased synovitis score, OARSI score, and the MMP13 expression in OA knee joints. In addition, the (AG‐P_m_)_10_‐OC_10_ also influenced macrophage polarization, increasing CD206 expression and decreasing iNOS levels. Taken together, the biocompatible (AG‐P_m_)_10_‐OC_10_ hydrogels show great potential for application in OA treatment and provide novel insights for the design of biomaterials in clinical practice.

## 4 Conclusion

In summary, a dynamic reversible cross‐linked hydrogel, (AG‐P_m_)_10_‐OC_10_, is fabricated via a Schiff‐base reaction as a functional platform for OA treatment. Specifically, P_m_ is synthesized through esterification and RAFT polymerization, and subsequently incorporated into the hydrogel, which exhibits significant anti‐inflammatory activity by regulating glycolysis in FLSs, and in turn, macrophage polarization. The biocompatible (AG‐P_m_)_10_‐OC_10_ demonstrates enhanced mechanical and self‐healing properties due to its covalent binding networks. Furthermore, (AG‐P_m_)_10_‐OC_10_ shows promise in attenuating OA progression, potentially linking to the downregulation of the YAP1/GLUT1 axis in the synovium. This study highlights the anti‐inflammatory capabilities of (AG‐P_m_)_10_‐OC_10_ through the integration of Bai, offering a novel and effective approach for OA therapy.

## 5 Experimental Section

### Synthesis of P_m_

Bai (0.12 mmol, 53.5 mg) and HEA (4.8 mmol, 556.8 mg) were dissolved in pyridine and cyclohexane (1: 1, v/v, 4 mL) with molecular sieves (10 wt%) and Novozym 435 (0.16 g) at 50 °C for 120 h. Then, the crude product (HEA‐Bai) was centrifuged, while CDSPA (48 µmol, 19.2 mg) and AIBN (9.6 µmol, 1.6 mg) were added into the supernatant with a feed ratio of monomer as CDSPA: AIBN = 100: 1: 0.2 for reversible addition‐fragmentation chain transfer (RAFT) polymerization. Subsequently, the mixture was degassed with nitrogen for 20 min and placed in a preheated oil bath at 60 °C. After 24 h, the crude was precipitated into diethyl ether, washed with distilled water, and dried at 40 °C vacuum condition to give Bai‐based polymer (P_m_).

### Drug Release Kinetics of P_m_

The drug release of Bai in P_m_ was conducted by dialysis bag method. P_m_ (15 mg) dissolved in DMSO (3 mL) was introduced into a dialysis bag immersed in PBS (pH 6.8 and 7.4) solution (27 mL), which was stirred at ambient temperature. Once upon the defined time intervals (0, 1, 5, 10, 24, 48, 72, and 96 h), 3 mL released solution was withdrawn and added an equivalent fresh medium into it. Bai content was determined by UV‐vis spectroscopy with the absorbance at 276 nm.

### Preparation of AG

Briefly, Gel (1.0 g) and ADH (4.0 g) were suspended in distilled water (100 mL). Then, HoBt (1.3 g) and EDC (1.9 g) were dissolved in DMSO/H_2_O (1: 1, v/v, 10 mL) mixture solvent and added into the former solution, while the pH value was adjusted to 5.0 by diluted hydrochloric acid. The aminated gelatin (AG) was collected at 25 °C after 24 h, which was dialyzed in distilled water for 24 h and lyophilized. The amount of free amino groups of AG was calculated by the ninhydrin assay according to the standard curve of glycine. The absorbance of AG at 570 nm was recorded by UV–vis spectroscopy.

### Preparation of P_m_‐Modified AG

P_m_ in DMSO (0.3 g, 6 mL) was dropwise mixed with AG aqueous solution (0.5 g, 50 mL). Then, EDC (0.25 g), and NHS (0.15 g) were added into the mixture at pH 6, and stirred at 37 °C for 24 h. The product was dialyzed in distilled water for 24 h and lyophilized.

### Preparation of OC

Sodium periodate (SP) aqueous solution (1.9 g, 20 mL) was dropwise added into ChS aqueous solution (5.0 g, 80 mL) at 25 °C. After 6 h stirring in the dark, ethylene glycol (5 mL) was used to neutralize extra SP. Thus, OC was dialyzed in distilled water for 24 h and lyophilized. ^1^H NMR was carried out to determine the degree of oxidation (DO) of ChS by the equation: DO = A_1.4_/(3×A_1.9_), while A_1.4_ and A_1.9_ represent the integrals of protons at δ = 1.4 and 1.9 ppm, respectively.

### Fabrication of (AG‐P_m_)‐OC

Lyophilized AG (0.1 g) was suspended in PBS solution (500 µL, pH 7.4) marked as (AG‐P_m_)_10_, while OC (10 wt%) solution was also prepared (OC_10_). Thus, the functional hydrogel was obtained by mixing (AG‐P_m_)_10_ and OC_10_ in equal volumes (1:1) according to the Schiff‐base reaction, which was denoted as (AG‐P_m_)_10_‐OC_10_. Similarly, (AG‐P_m_)_5_‐OC_10_ was also fabricated by the steps above.

### Rheological Study

The rheological study of the functional hydrogels was carried out on a Malvern Kinexus Pro+ rheometer with 20 mm parallel plates at 25 °C by four modes (gap = 1.0 mm). 1) Strain amplitude sweep mode was implemented from 0.1 to 300% strain to determine the linear viscoelastic region for the hydrogels, which set 6.28 rad s^−1^ as a constant frequency. 2) Alternating strain amplitude sweep mode was carried out to evaluate the self‐healing properties of the hydrogels. Particularly, 1% and 300% strain were the minimum and maximum values for (AG‐P_m_)_10_‐OC_10_, while 1% and 200% strain were for (AG‐P_m_)_10_‐OC_10_. Each run had a 100 s interval. 3) Frequency sweep mode was performed ranging from 0.1 to 300 rad/s to assess the stability of the hydrogels. 4) Shear‐thinning test mode was used to reveal the viscosity properties of the hydrogels ranging from 0.1 to 300 s^−1^.

### Mechanics Performance Test

The mechanical properties of the hydrogels were detected by a Universal Testing Machine (Bose ELF3200). The compressive strength tests were performed on cylinder‐shaped samples (diameter = 6 mm, height = 12 mm). The compression started from the original height to 50% strain. In the tensile tests, the tension speed was 1 mm min^−1^.

### Friction Test

The friction tests were performed on a Rtec MFT‐5000 tribometer (Rtec Instruments, Inc., USA). All tests were carried out in PBS solution (pH 6.8), and a glass ball was chosen as the tribo‐pair. The tests were conducted by 5 N and 1 Hz for 500 s.

### Swelling Behavior

The lyophilized hydrogels (50 mg) were immersed in PBS (pH 7.4, 5 mL) at 37 °C. Then the hydrogels were dropped out at pre‐set stages (1, 2, 4, 8, 12, 24, and 48 h). After centrifugation, a filter paper was used to remove excess moisture. The swelling ratio (SR) was determined by the following equation: SR = (W_S_‐W_0_)/W_0_ × 100%. W_S_ and W_0_ were marked as the weights of swollen and initial lyophilized hydrogels, respectively.

### Degradation Performance

The lyophilized hydrogels (0.1 g) were immersed in 10 mL PBS (pH 6.8 and 7.4) or collagenase I (0.5 U mL^−1^) solutions for hydrolysis and enzymatic degradation. The solutions were stirred at room temperature and the hydrogels were dropped out at pre‐set stages, 72, 96, 120, and 144 h for hydrolysis, 12, 24, 36, 48, 60, and 72 h for enzymatic degradation in detail. After lyophilization, the degradation rate (DR) was determined by the following equation: DR = (W_0_–W_t_)/W_0_ × 100%. W_t_ and W_0_ were marked as the weights of degradation and initial lyophilized hydrogels, respectively.

### Bai Releasing from the Functional Hydrogel

(AG‐P_m_)_10_‐OC_10_ was placed in PBS solution (pH 7.4 or 6.8) at 35 °C. Samples (3 mL) were taken out at pre‐set intervals (0, 12, 24, 72, 120, and 168 h) after 10 min centrifugation. Bai release from (AG‐P_m_)_10_‐OC_10_ was investigated by UV–vis spectroscopy with the absorbance at 276 nm, according to the standard curve of Bai.

### Cell Culture

Investigations involving human participants obtained informed consent from all participants after explaining the nature and possible consequences of the studies. The study protocol was reviewed and approved by the Ethics Committee of the First Affiliated Hospital of Jinan University (Ethical approval number: KY‐2021‐065). Human fibroblast‐like synoviocytes (FLSs) were obtained by synovial tissues sliced into small pieces with enzymatic digestion (0.2% Collagenase Type I) at 37 °C for 2 h shaking (90 rpm). After centrifugation, FLSs were collected and cultured in Dulbecco's modified Eagle's medium (DMEM) with 10% FBS, 100 U mL^−1^ penicillin, and 100 µg mL^−1^ streptomycin. RAW264.7 cells were cultured in DMEM with 10% FBS, 100 µg mL^−1^ streptomycin, and 100 U mL^−1^ penicillin for the next experiments.

### Cellular Uptake Assay

Cellular uptake efficiency and intracellular distribution of P_m_ in FLSs and RAW264.7 cells were examined using confocal laser scanning microscopy (CLSM) (Zeiss 880) and quantified using fluorescence‐activated cell sorting (FACS) (BD LSRFortessa). Initially, P_m_ was labeled with 1,1′‐dioctadecyl‐3,3,3′,3′‐tetramethylindodicarbocyanine (DiD). Subsequently, FLSs and RAW264.7 cells were individually plated in confocal dishes and exposed to different concentrations of P_m_ (5, 10, 15, and 17.5 µg mL^−1^) for various durations (4, 8, 12, 16 h). Following fixation with 4% paraformaldehyde (PFA) and permeabilization with 0.1% Triton, the cell nucleus and cytoskeleton were stained with DAPI (0.5 µg mL^−1^, CST, 0483, USA) and Actin‐Tracker Green‐488 (Beyotime, C2201S, China), preceding quantitative analysis.

### RNA Extraction and Quantitative Real‐Time PCR (qRT‐PCR)

Total RNA was extracted from FLSs and RAW264.7 cells utilizing TRIzol Plus (Takara Technologies, D9108A, Shiga, Japan) following the manufacturer's guidelines. The first‐strand cDNA was synthesized through reverse transcription employing a Synthesis SuperMix Kit (TransGen, AQ211, China). Primers for detecting *IL‐1β*, *IL‐6*, *IL‐8*, and *iNOS* are detailed in Table S1 (Supporting Information). qRT‐PCR was conducted through One Step Real‐Time PCR (Applied Biosystems) using Fast SYBR GREEN Master Mix (Life Technologies). Each qRT‐PCR assay was duplicated across a minimum of three independent trials, and the gene expressions were normalized to GAPDH utilizing the conventional 2^−ΔΔCt^ method.

### Anti‐Inflammatory Activity

FLSs were plated in 6‐well plates and exposed to Bai or P_m_ at a concentration of 15 µg mL^−1^ for 6, 12, and 24 h, followed by stimulation with IL‐1β (10 ng mL^−1^) for 6 h. qRT‐PCR was performed to investigate the anti‐inflammatory activity of P_m_ by the expression levels of *IL‐1β*, *IL‐6*, and *IL‐8*.

### Seahorse XF96 Glycolysis Analysis

Extracellular Acidification Rate (ECAR) and Oxygen Consumption Rate (OCR) were assessed using a Seahorse XF96 extracellular flux analyzer (Seahorse Bioscience in Billerica, Massachusetts, USA). In short, FLSs were seeded in Seahorse XF96 culture plates at a density of 1 × 10^4^ per well and incubated with 10% FBS DMEM for 24 h. Subsequently, they were treated with various concentrations of glucose with or without Bai or P_m_ for another 24 h. The experimental protocol adhered to methods outlined in previous studies.^[^ ^6^ ^]^ All Seahorse flux data were normalized based on cell counts obtained using a cell counter machine.

### Western Blotting Analysis

The primary antibodies, anti‐YAP1 antibodies (1:1000, CST, 14 074, USA) and anti‐GAPDH antibodies (1:1000, CST, 2118, USA), were removed after an overnight incubation. Then, anti‐rabbit IgG HRP‐conjugated antibodies (1:1000, CST, 7074, USA) were incubated for 1 h at room temperature. Protein detection was performed on the Western ECL Substrate (Bio‐Rad, 170–5061, USA) and the Enhanced Chemiluminescence Western Blot System (Tanon 5200, Tanon, China).

### Osteoarthritis (OA) Model and In Vivo Imaging

All procedures involving laboratory animals were conducted in accordance with institutional guidelines for the care and use of laboratory animals. Our research protocol was reviewed and approved by the IACUC of Jinan University (Ethical approval number: IIACUC‐20230821‐02). Eight‐week‐old C57BL/6 mice were purchased from GemPharmatech and primed for OA mice through anterior cruciate ligament transection (ACLT) under pentobarbital sodium anesthesia (40 mg kg^−1^). To ensure consistency, the OA mice were randomly divided into three groups, each containing three mice. Following this, Bai, P_m_, and (AG‐P_m_)_10_‐OC_10_ (20 mg kg^−1^) labeled with 1,1‐dioctadecyl‐3,3,3,3‐tetramethylindotricarbocyanine iodide (DiR, 0.5 mg mL^−1^) were injected into the left knee joint cavity. Subsequently, the mice were monitored at specific intervals (0, 1, 4, 7, 10, and 14 days) using an imaging system (Tanon ABL X5) to assess the joint retention situation post intra‐articular administration. For biodistribution analysis, OA mice were evaluated 24 h after an intra‐articular injection with DiR‐labeled Bai, P_m_, and (AG‐P_m_)_10_‐OC_10_. Major organs (heart, liver, spleen, lung, and kidney), blood samples, and joint tissues (surrounding tissue, femoral condyle, tibial plateau, and tibiofibula) were harvested and subjected to fluorescent visualization scans.

### Histological Assessment for OA Treatment

The OA mice were treated with intra‐articular injection every 10 days to investigate the therapeutic effect of Bai, P_m_, and (AG‐P_m_)_10_‐OC_10_. The mice were sacrificed after 6 weeks, and their ipsilateral L3‐L5 dorsal root ganglia (DRG), knee joints, and major organs were collected and fixed with 4% PFA for 1 day. The joints were dealt with EDTA (0.5 mol L^−1^, pH 7.4) for 3 weeks to decalcify and sliced into paraffin or frozen sections. Sucrose (20%) and polyvinylpyrrolidone (PVP, 2%) were utilized to dehydrate tissues to prepare frozen sections which were embedded by the mixture of sucrose (20%), PVP (2%), and gelatin (8%). The frozen sections of knee joints at 60 µm and the paraffin sections at 6 µm were used for the following study.

For immunofluorescence (IF) staining, anti‐GLUT1 (1:100, CST, 12 939, USA), anti‐TRPA1 (1:100, Novus, NB110‐40763, USA), anti‐iNOS (1:100, Santa Cruz, sc7271, USA), and anti‐CD206 (1:400, CST, 24595S, USA) were individually incubated with paraffin sections overnight at 4 °C, and subsequently combined with secondary antibodies, Alexa Fluor 488‐conjugated (1:200, CST, 4408S, USA) or Alexa Fluor 555‐conjugated (1:200, CST, 4413S, USA). Meanwhile, DAPI (0.5 µg/mL, CST, 0483, USA) was prepared to locate the nucleus. Additionally, frozen sections incubated with anti‐PDGFR‐α (1:100, Santa‐Cruz, sc‐398206, USA) were subjected to the TSAPlus Kit according to the protocols, and the images were obtained by a laser confocal microscope (Zeiss 880).

For immunohistochemical (IHC) staining, paraffin sections that underwent deparaffinization and antigen retrieval were incubated with anti‐MMP13 (1:100, Servicebio, GB11247, China) overnight at 4 °C. Then, the samples were incubated with secondary antibodies after 3 times washing, which were further color‐developed by DAB solution (CST, 8509P, USA). The results were acquired by a light microscope (390335, Leica, Germany) and evaluated by ImageJ software (NIH, USA). Typically, sagittal paraffin sections as 6 µm were conducted to H&E (G1004; G1001, Servicebio, China) and S/O staining (G1053, Servicebio, China) to assess the biocompatability and the OA development according to the Osteoarthritis Research Society International (OARSI) score and Synovitis score, respectively.

### Gait Analysis

A gait analysis system (CatWalk XT, Noldus) was utilized to detect stride length and single stance of OA mice, which accepted ACLT surgery after 6 weeks. Each mouse was trained to walk through a transparent Plexiglass track, and their gait was recorded using a high‐speed camera.

### Macrophage Polarization

RAW264.7 cells were cultured in 6‐well plates and treated with Bai or P_m_ (15 µg mL^−1^) for 6, 12, and 24 h, followed by stimulation with LPS (100 ng mL^−1^) for 6 h. Then, qRT‐PCR analysis was conducted on RAW264.7 cells to evaluate expression levels of *iNOS*, determining M1 macrophage polarization. Moreover, IF images of RAW264.7 cells under different treatments were investigated. The pre‐treated RAW264.7 cells were fixed by 4% PFA and permeabilized with 0.1% Triton X‐100. The cells were washed 3 times with PBS and stained with anti‐iNOS (1:200, Santa Cruz, sc7271, USA), DAPI (0.5 µg mL^−1^, CST, 0483, USA), and Actin‐Tracker Green‐488 (Beyotime, C2201S, China). Images were obtained by a laser confocal microscope (Zeiss 880).

### Statistical Analysis

Unless otherwise specified, all experiments were conducted three times independently. Data are shown as the means ± standard deviations and analyzed via Student's *t*‐test for two groups and One‐way ANOVA for multiple groups, followed by Tukey's test. The differences were statistically significant as ^*^ *p* < 0.05, ^**^ *p* < 0.01, and ^***^ *p* < 0.001.

## Conflict of Interest

The authors declare no conflict of interest.

## Author Contributions

Y.Y., Q.H., and Q.S. contributed equally to this work. Y.Y., Q.H., and Q.S. performed data curation, investigation, visualization, and writing – original draft. Y.P., B.Y., F.L., and J.C. performed the investigation. C.X., Z.L., M.T., Z.J., and R.Z. performed formal analysis. Z.Z. performed supervision. F.X. performed supervision and writing review & editing. H.T.Z. performed conceptualization, supervision, writing – original draft, and writing review & editing.

## Supporting information



Supporting Information

Supplemental Video 1

## Data Availability

The data that support the findings of this study are available from the corresponding author upon reasonable request.
