# Supplementary material for: A Baicalin‐Based Functional Polymer in Dynamic Reversible Networks Alleviates Osteoarthritis by Cellular Interactions
Source: Adv Sci (Weinh). 2025 Jan 22;12(10):2410951. doi: 10.1002/advs.202410951 (PMC11904974; doi:10.1002/advs.202410951)
Supplement: Supplementary file 1 — Supporting Information [file ADVS-12-2410951-s002.docx]

**Supporting Information**

**A Baicalin-based Functional Polymer in Dynamic Reversible Networks Alleviates Osteoarthritis by Cellular Interactions**

*Yili Yang, Qinxiao Hu, Qingfeng Shao, Yachen Peng, Bo Yu, Fangji Luo, Jiajing Chen, Chenhao Xu, Zhenyan Li, Manseng Tam, Zhenyu Ju, Ronghua Zhang, Feiyue Xing*,* *Zhengang Zha*,* *Huan-Tian Zhang**

**Keywords:** Osteoarthritis, Bai-based polymer, Schiff-base hydrogel, anti-inflammation, glycolysis

**This PDF file includes:**

Supplementary Text: Experimental Section

Figure S1 to S13

Figure S1. Standard curve of baicalin (Bai)

Figure S2. ^1^H NMR spectra of amination of gelatin (AG)

Figure S3. Standard curve of glycine

Figure S4. ^1^H NMR spectra of oxidation of chondroitin sulfate (OC)

Figure S5. SEM images of the Schiff base hydrogel

Figure S6. Rheological study of (AG-P_m_)_5_-OC_10_

Figure S7. Cytotoxicity of P_m_ in FLSs and RAW264.7 cells

Figure S8. Seahorse analysis of OCR experiments

Figure S9. Molecular docking study of Bai to epidermal growth factor receptor (EGFR)

Figure S10. Western Blotting analysis of P_m_ to yes-associated protein (YAP1)

Figure S11. Biocompatibility of (AG-P_m_)_10_-OC_10_

Figure S12. Bai-based formulations suppress the GLUT1 expression

Figure S13. Images of all samples of the formulation’s treatment effects on OA mice

Tables S1

Table S1. The primers used for quantitative RT-PCR

**Supplementary Text**

**Experimental Section**:

*Materials*: Unless otherwise specified, all chemicals were reagent grade and were used as received. Baicalin (Bai, 90%), chondroitin sulfate (ChS, 85%), adipic acid dihydrazide (ADH, 98%), *tert*-Butyl carbazate (*t*-BC, 98%), 1-hydroxybenzotriazole hydrate (HoBt, 97%), ninhydrin (95%), glycine (99.5%), sodium periodate (SP, 99.5%), and 1,1-dioctadecyl-3,3,3,3-tetramethylindotricarbocyanine iodide (DiR) were purchased from Shanghai Macklin Biochemical Co., Ltd. Novozym 435 was purchased from Novozymes A/S. Collagenase I was purchased from Shanghai Acmec Biochemical Co., Ltd. 2-Hydroxyethyl acrylate (HEA, 97%), 2,2'-azobis(2-methylpropionitrile) (AIBN, 98%), ethyl-dimethyl-aminopropylcarbodiimide (EDC, 98%), and *N*-hydroxy-succinimide (NHS, 98%) were purchased from Shanghai Aladdin Bio-Chem Technology Co., Ltd. 4-Cyano-4-[[(dodecylthio)carbonothioyl]thio]pentanoic acid (CDSPA, 95%) was purchased from Bide Pharmatech Co., Ltd. Gelatin (Gel, type A, CP) was purchased from Sigma-Aldrich. Cell Counting Kit-8 (CCK8), 1,1′-dioctadecyl-3,3,3′,3′-tetramethylindodicarbocyanine, 4-chlorobenzenesulfonate salt (DiD), and Actin-Tracker Green-488 were purchased from Beyotime Biotechnology. IL-1β, IL-6, and IL-8 were purchased from PeproTech. Anti-YAP1, anti-GAPDH, anti-rabbit IgG HRP-conjugated antibodies, anti-GLUT1, DAB solution, DAPI, Alexa Fluor 488-conjugated antibodies, and Alexa Fluor 555-conjugated antibodies were purchased from CST. Anti-PDGFR-α, anti-CD206, and anti-iNOS were purchased from Santa-Cruz. Anti-TRPA1 was purchased from Novus. Anti-MMP13, hematoxylin-eosin (H&E), and safranin O/fast green (S/O) were purchased from Servicebio.

*Instruments:* High performance liquid chromatography (HPLC) was performed on Agilent 1260 using binary solvent (methanol: water = 85: 15, v/v) with phosphoric acid (0.1%) as eluents (0.9 mL/min). High resolution mass spectroscopy (HRMS) was carried out on X500R QTOF system with the positive ionization mode. Nuclear magnetic resonance (NMR) spectra were recorded on a Bruker 300 MHz spectrometer at 25 ℃. Size exclusion chromatography (SEC) was conducted on an Agilent 1260 Infinity II at 35 ℃ with THF as an eluent (1.0 mL/min). Ultraviolet-visible (UV-vis) spectra were recorded on a Techcomp UV1000 spectrophotometer and Fourier transform infrared (FT-IR) spectra were obtained on a Bruker Vertex70 spectrometer within the range from 400 to 4000 cm^–1^. Field emission scanning electron microscope (SEM) analysis was performed on ULTRA 55 (Zeiss). The rheological performance of the hydrogel adhesives were studied on a Malvern Kinexus Pro+ rheometer (gap = 1.0 mm) using 20 mm parallel plates at 25 ℃. CCK8 assay was performed on a microplate reader (BioTek Synergy H1) by recording the absorbance at 450 nm. The cellular uptake and immunofluorescence staining were carried out on a confocal laser scanning microscopy (CLSM) (Zeiss 880). *In vivo* fluorescence visualized images were obtained by an imaging system (Tanon ABL X5). Immunohistochemical staining images were visualized by a light microscope (Leica). Extracellular acidification rate (ECAR) of glycolysis analysis was measured by an XF96 extracellular flux analyzer (Seahorse Bioscience).

*Cytotoxicity Study*: FLSs and RAW264.7 cells were seeded in 96-well plates (3 × 10^3^/well) and placed at 37 ℃ (5% CO_2_), respectively. P_m_ were added into two kinds of cells at five different concentrations (5, 10, 15, 20, and 25 μg/mL) related to an equivalent dose of Bai, while cells in fresh medium with no addition of P_m_ were negative control groups. After 24 h, the culture medium was removed and cells were washed with PBS. Then, CCK-8 solution was added for 2 h incubation and the absorbance was recorded at 450 nm by a microplate reader (BioTek Synergy H1).

*Molecular Docking Study*: For processing EGFR, the crystal structures of EGFR complex (PDB ID: 7SZ0) for molecular docking were downloaded from Protein Data Bank (https://www.rcsb.org/, accessed on 21 August 2024). Prior to molecular docking, the structures of EGFR complex were prepared using the protein preparation wizard module of software (Release 2019-2, Schrödinger LLC, New York, NY, USA). The protein preparations, including protonation-state adjustment, water removal, disulfide bonds, hydrogen atom and missing heavy atom addition, and structural minimization, were performed by the Maestro module of Schrödinger software.

For processing Bai, the crystal structure was obtained from PubChem database (https://pubchem.ncbi.nlm.nih.gov, accessed on 21 August 2024). The LigPrep panel in Maestro was applied to ligand preprocessing, including (I) an OPLS_2005 force field, (II) no change for ionization, (III) a desalt option, (IV) chirality determination from the 3D structure, (V) the generation of one low energy conformer at most per ligand, and (VI) an output in SDF format. The generated 3D conformer of Bai was subjected to next investigation.

For molecular docking, the molecular docking parameter in the EGF binding site of EGFR was determined by redocking the substrate EGF into EGFR, respectively, to resume the binding mode and interaction of EGF in the co-crystal structure of EGFR (PDB ID: 7SZ0). Consequently, the receptor grid was set to be centered on the reference ligand and the grid size is similar to that of it. All other parameters were kept as the default in Schrödinger with the Glid Standard Precision (SP) mode.

For calculating binding energy, Schrödinger Prime MM-GBSA module was used to calculate the relative binding-free energy (ΔG bind) of screened EGFR with Bai using the equation ΔGGB = ΔEMM+ΔGsolv + ΔGSA. The ΔGGB is the electrostatic solvation energy. ΔEMM is the difference between the minimized energies of the protein-ligand complex and the total energies of the protein and ligand in free form. ΔGsolv is the difference in the GBSA solvation energies of the protein-ligand complex and the sum of the solvation energies of protein and ligand in free form. ΔGSA is the difference in the surface area energies for an unbound form of protein and ligand.


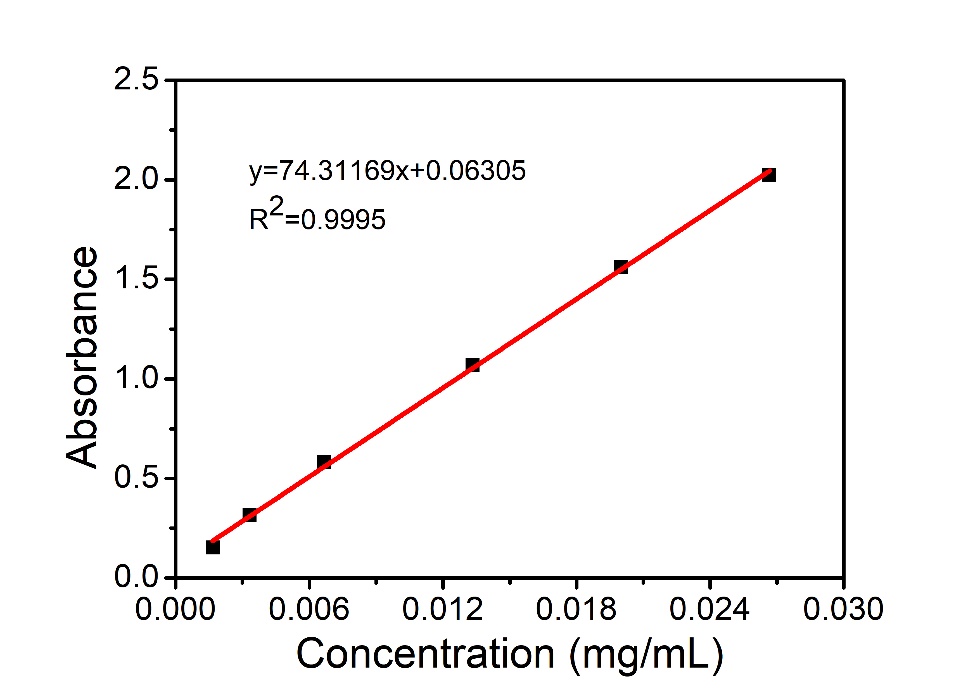


**Figure S1.** Standard curve of Bai by UV-vis spectra at 276 nm.


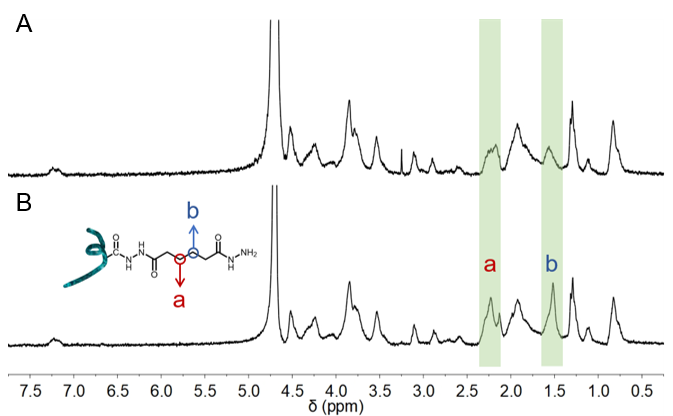


**Figure S2.** ^1^H NMR spectra of A) Gel and B) AG (300 MHz, D_2_O).


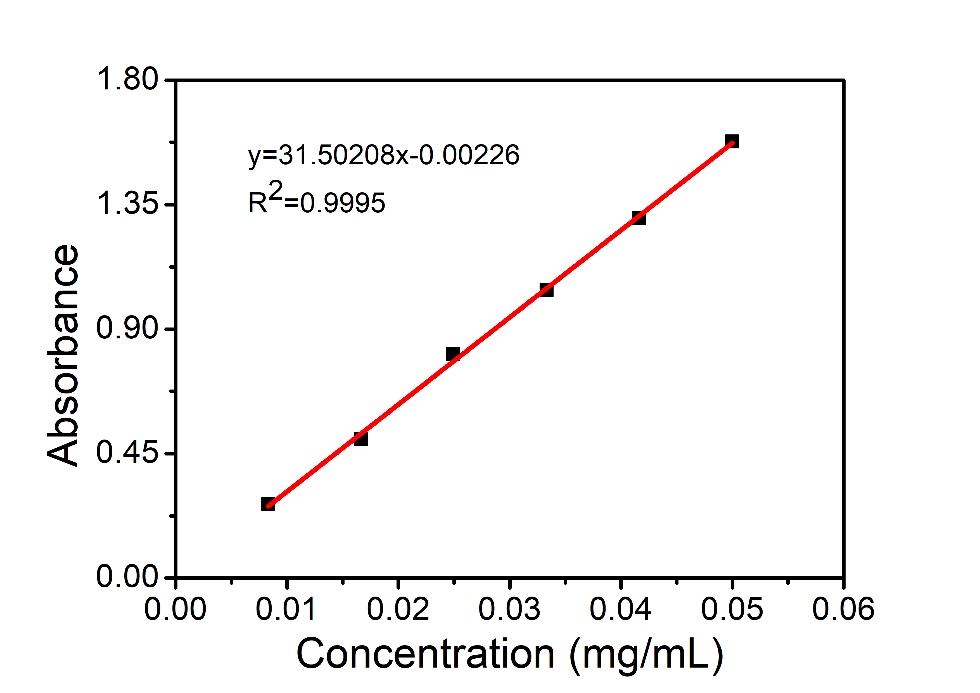


**Figure S3.** Standard curve of glycine at 570 nm.


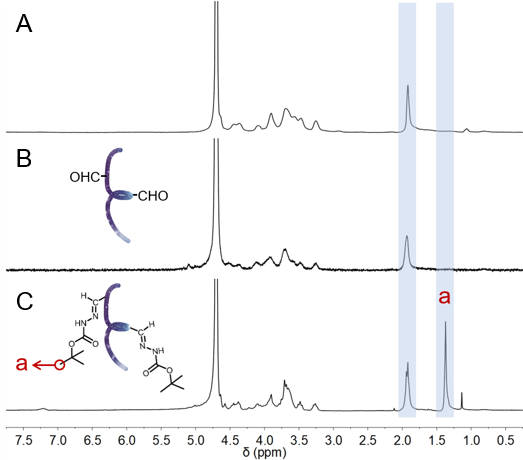


**Figure S4.** ^1^H NMR spectra of A) ChS, B) OC, and C) *t*-OC (300 MHz, D_2_O).


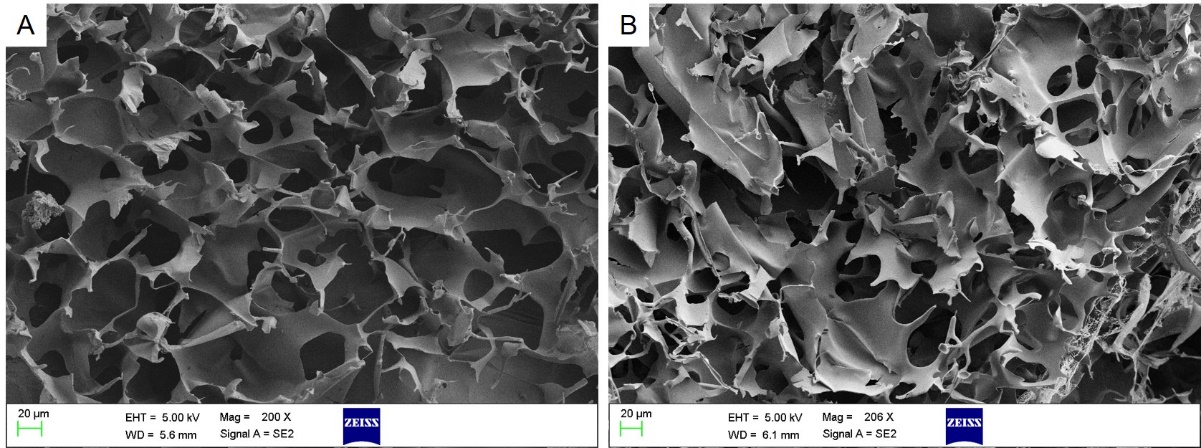


**Figure S5.** SEM images of A) (AG-P_m_)_5_-OC_10_ and B) (AG-P_m_)_10_-OC_10_.


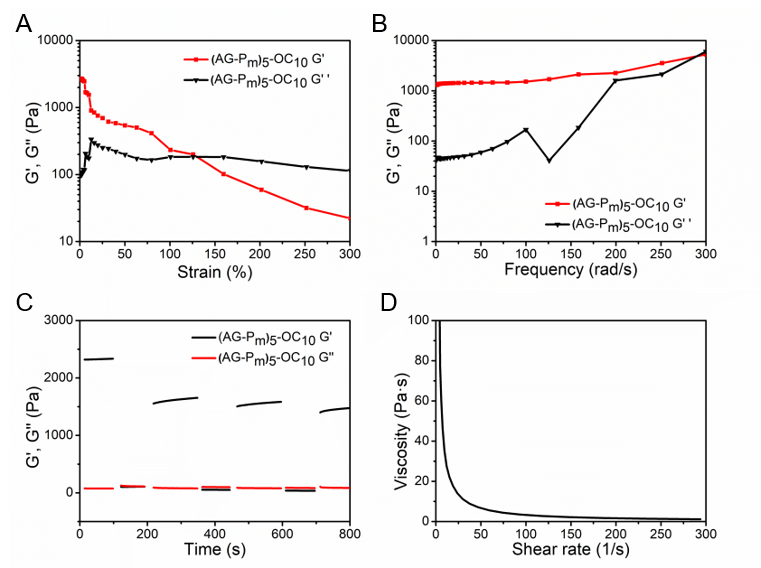


**Figure S6.** Rheological study of (AG-P_m_)_5_-OC_10_. A) Strain amplitude sweep mode. B) Frequency amplitude sweep mode. C) Alternating strain amplitude sweep mode. D) Shear thinning test.


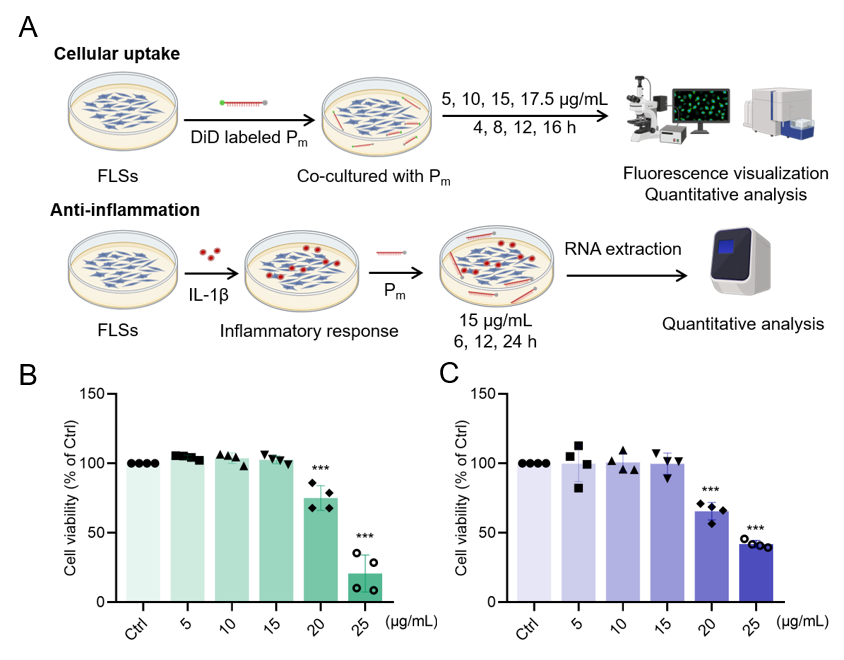


**Figure S7.** Bioactive assessments of P_m_ *in vitro*. A) Schematic of cellular uptake and anti-inflammatory activity of P_m_ to FLSs. B) Cytotoxicity of P_m_ in FLSs. C) Cytotoxicity of P_m_ in RAW264.7 cells. Data are presented as mean ± SD, ****p* < 0.001, and Student’s t-test for (B,C).


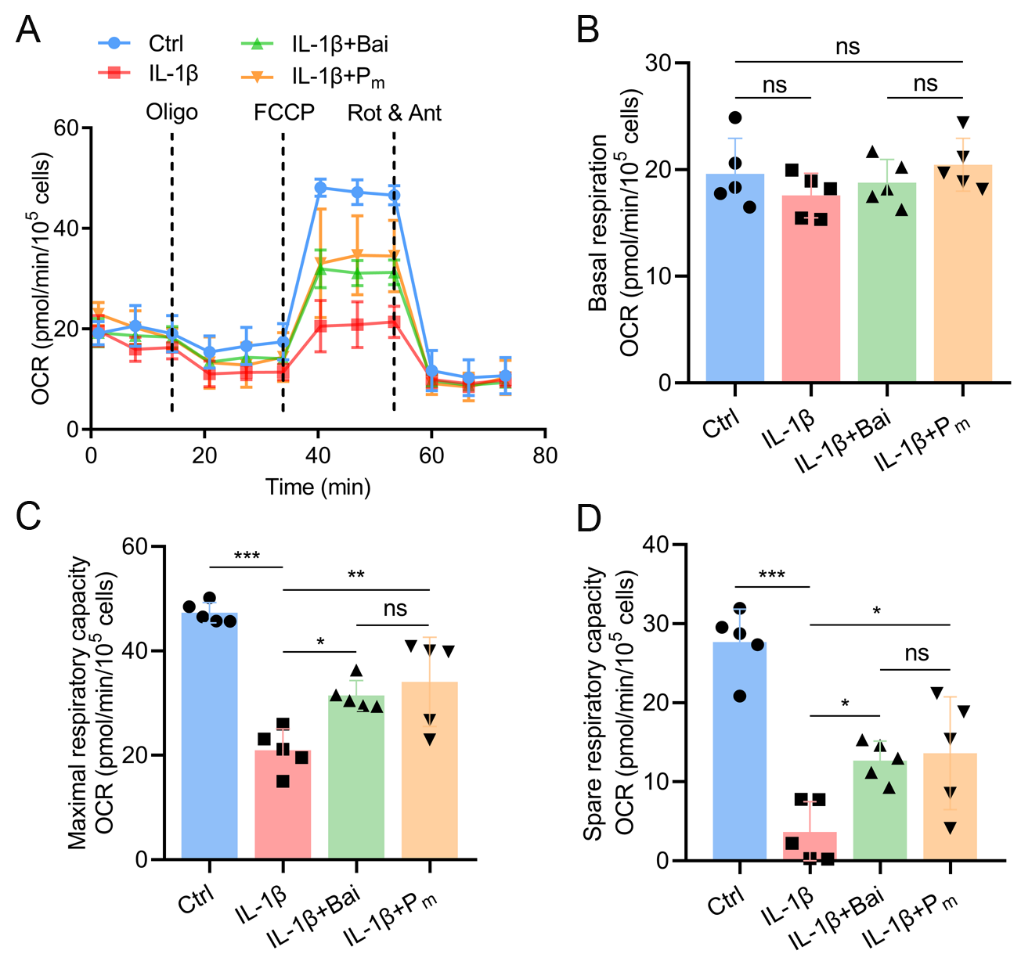


**Figure S8.** Seahorse metabolic flux measuring A) OCR, B) basal respiration, C) maximal respiratory capacity, and D) spare respiratory capacity in FLSs treated with Bai and P_m_ in the presence or absence of IL-1β for 24 h. Data are presented as mean ± SD. n.s.: not significant, **p* < 0.05, ***p* < 0.01, and ****p* < 0.001. One-way ANOVA for (B-D).


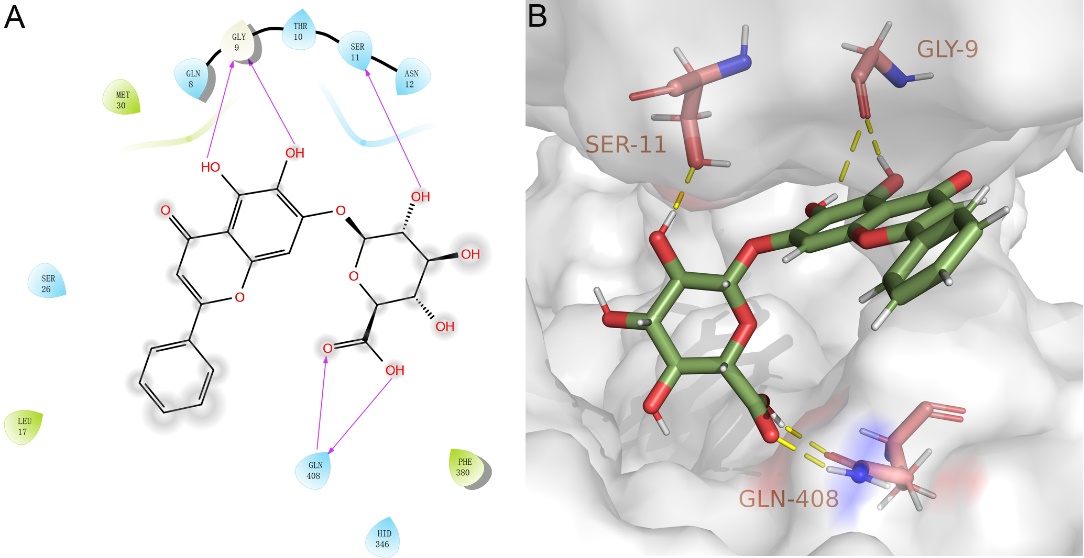


**Figure S9.** Molecular docking study of Bai to epidermal growth factor receptor (EGFR). A) 2D and B) 3D molecular docking modeling of EGFR residues around Bai.


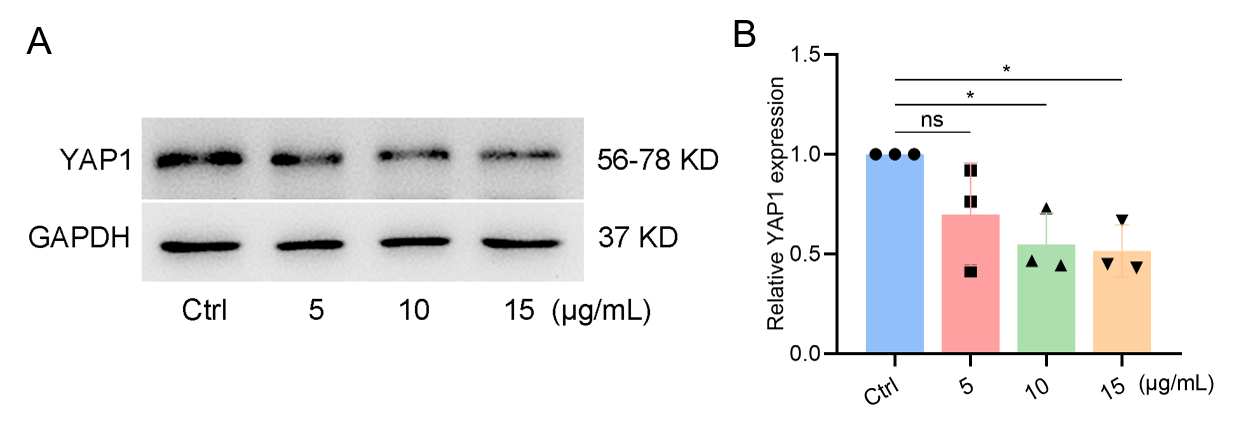


**Figure S10.** Western Blotting analysis of P_m_ (5, 10, and 15 μg/mL)) to yes-associated protein (YAP1). A) Immunoblotting of YAP1 expression in FLSs with P_m_ treatment (5, 10, and 15 μg/mL). B) Quantification analysis of YAP1 expression in (A). Data are presented as mean ±SD. n.s.: not significant, **p* < 0.05. One-way ANOVA for (B).


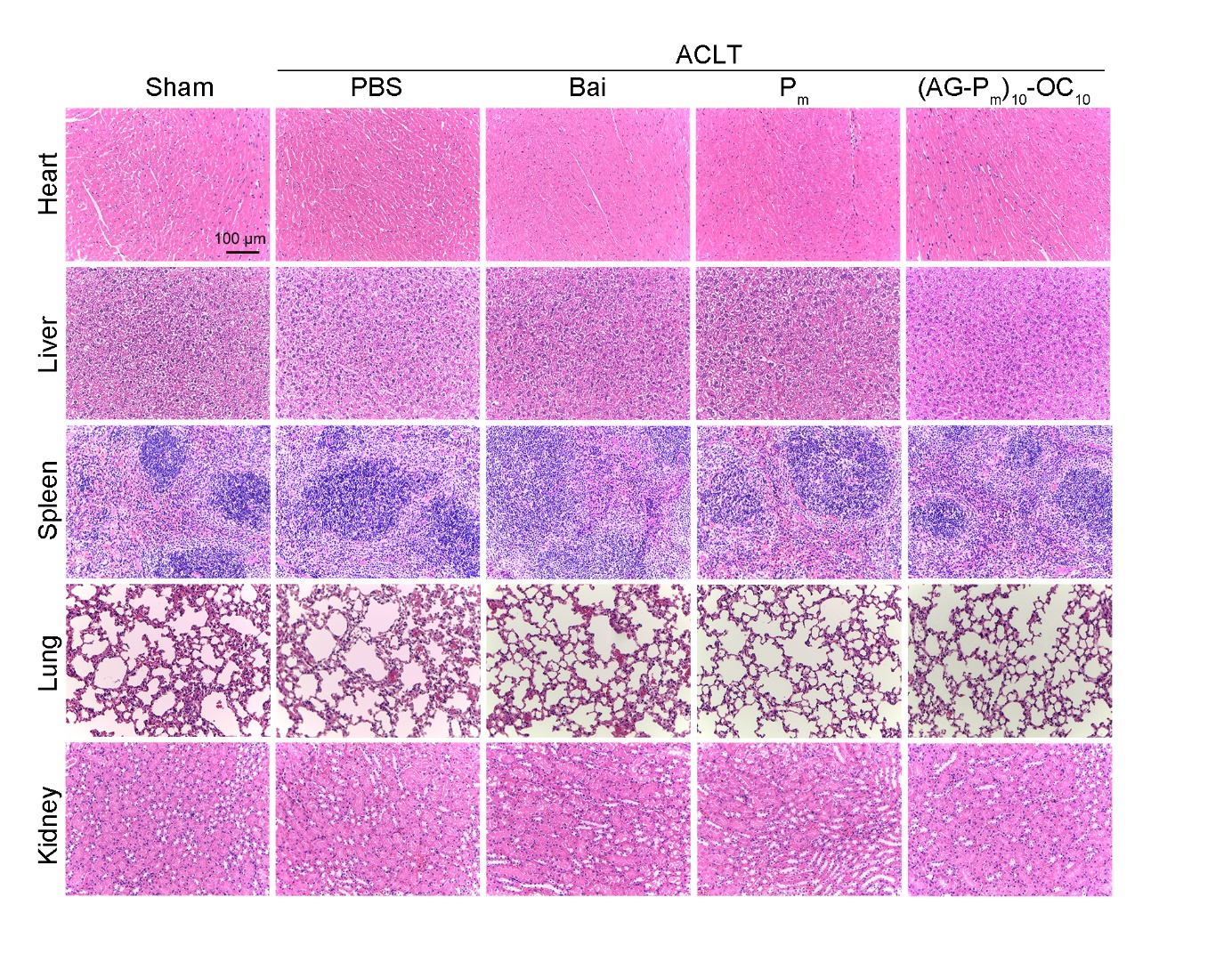


**Figure S11.** Biocompatibility of Bai-based formulations in OA mice.


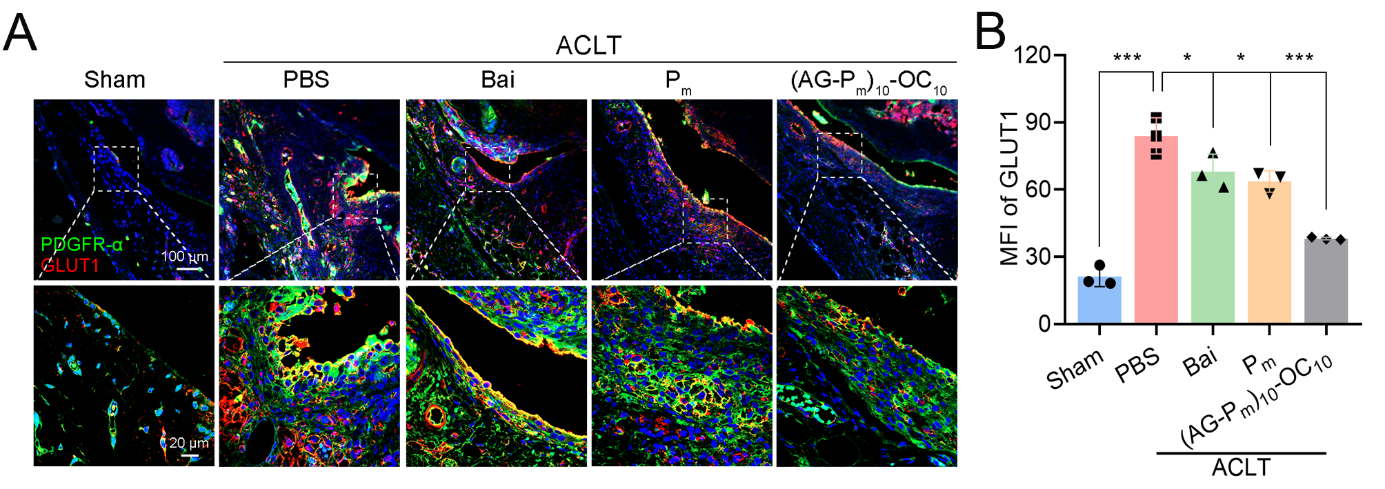


**Figure S12.** Bai-based formulations suppress the GLUT1 expression. A,B) Representative fluorescence images of GLUT1 (red) (A) and quantitative analysis of MFI of GLUT1 (B) in following treatments with Bai, P_m_, and (AG-P_m_)_10_-OC_10_ for 6 weeks. Scale bars = 100 and 20 μm.


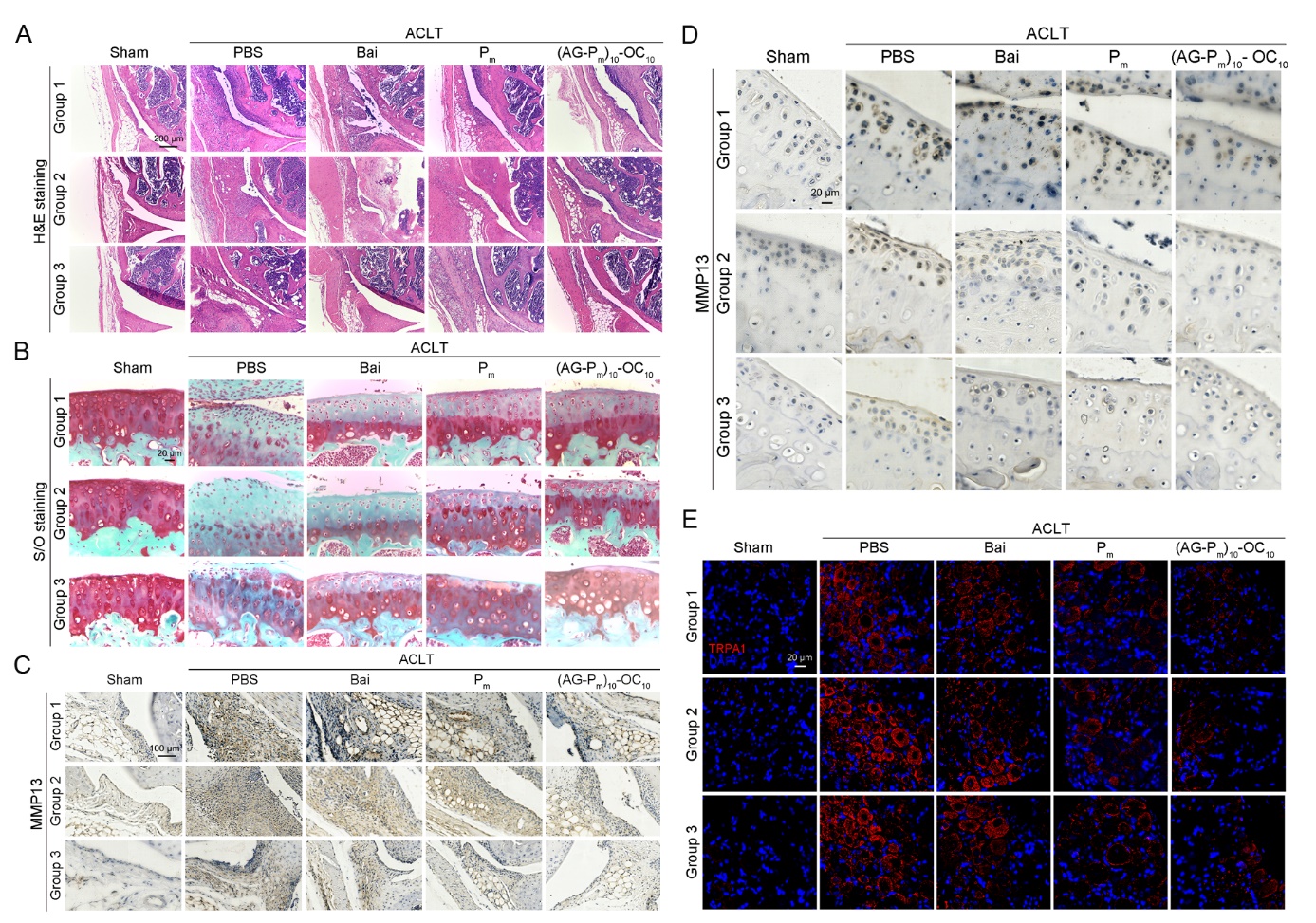


**Figure S13. Images of all samples of the formulation’s treatment effects on OA mice. A)** H&E staining images of OA model treated with Bai, P_m_, and (AG-P_m_)_10_-OC_10_ for 6 weeks. Scale bars = 20 μm. B) S/O staining images of OA model treated with Bai, P_m_, and (AG-P_m_)_10_-OC_10_ for 6 weeks. Scale bars = 20 μm. C) IHC images of MMP13 in synovial tissues of OA model treated with Bai, P_m_, and (AG-P_m_)_10_-OC_10_ for 6 weeks. Scale bars = 100 μm. D) IHC images of MMP13 in cartilage of OA model treated with Bai, P_m_, and (AG-P_m_)_10_-OC_10_ for 6 weeks. Scale bars = 20 μm. E) Fluorescence images of TRPA1 (red) in DRG tissues treated with Bai, P_m_, and (AG-P_m_)_10_-OC_10_ for 6 weeks. Scale bars = 20 μm. (The images of Group 1 are presented in the text as representative images.)

**Table S1.** The primers used for quantitative RT-PCR

| **Genes** | **Forward** | **Reverse** |
| --- | --- | --- |
| **Human** |  |  |
| *GAPDH* | CTGACTTCAACAGCGACACC | CCCTGTTGCTGTAGCCAAAT |
| *IL-1β* | ACTCAAGAATGGGCGGAAAGC | TCAGGAACAGCCACCAGTGAG |
| *IL-6* | CACTGGTCTTTTGGAGTTTGAG | GGACTTTTGTACTCATCTGCAC |
| *IL-8* | CAGTTTTGCCAAGGAGTGCTA | GGTCCACTCTCAATCACTCTCAG |
| *YAP1* | CCCTCGTTTTGCCATGAACC | GTTGCTGCTGGTTGGAGTTG |
| **Mice** |  |  |
| *GAPDH* | TGTGTCCGTCGTGGATCTGA | TTGCTGTTGAAGTCGCAGGAG |
| *iNOS* | ACTACTACCAGATCGAGCCC | GCATGGAAGCAAAGAACACC |
